# Nirmatrelvir-resistant SARS-CoV-2 variants with high fitness in an infectious cell culture system

**DOI:** 10.1126/sciadv.add7197

**Published:** 2022-12-21

**Authors:** Yuyong Zhou, Karen Anbro Gammeltoft, Line Abildgaard Ryberg, Long V. Pham, Helena Damtoft Tjørnelund, Alekxander Binderup, Carlos Rene Duarte Hernandez, Carlota Fernandez-Antunez, Anna Offersgaard, Ulrik Fahnøe, Günther Herbert Johannes Peters, Santseharay Ramirez, Jens Bukh, Judith Margarete Gottwein

**Affiliations:** ^1^ Copenhagen Hepatitis C Program (CO-HEP), Department of Infectious Diseases, Copenhagen University Hospital–Hvidovre, 2650 Hvidovre, Denmark.; ^2^ CO-HEP, Department of Immunology and Microbiology, Faculty of Health and Medical Sciences, University of Copenhagen, 2200 Copenhagen, Denmark.; ^3^ Department of Chemistry, Technical University of Denmark, 2800 Kongens Lyngby, Denmark.

## Abstract

The oral protease inhibitor nirmatrelvir is of key importance for prevention of severe coronavirus disease 2019 (COVID-19). To facilitate resistance monitoring, we studied severe acute respiratory syndrome coronavirus 2 (SARS-CoV-2) escape from nirmatrelvir in cell culture. Resistant variants harbored combinations of substitutions in the SARS-CoV-2 main protease (Mpro). Reverse genetics revealed that E166V and L50F + E166V conferred high resistance in infectious culture, replicon, and Mpro systems. While L50F, E166V, and L50F + E166V decreased replication and Mpro activity, L50F and L50F + E166V variants had high fitness in the infectious system. Naturally occurring L50F compensated for fitness cost of E166V and promoted viral escape. Molecular dynamics simulations revealed that E166V and L50F + E166V weakened nirmatrelvir-Mpro binding. Polymerase inhibitor remdesivir and monoclonal antibody bebtelovimab retained activity against nirmatrelvir-resistant variants, and combination with nirmatrelvir enhanced treatment efficacy compared to individual compounds. These findings have implications for monitoring and ensuring treatments with efficacy against SARS-CoV-2 and emerging sarbecoviruses.

## Teaser

Specific SARS-CoV-2 main protease substitutions mediate high nirmatrelvir resistance and fitness in an infectious culture system.

## INTRODUCTION

Effective options for treatment of coronavirus disease 2019 (COVID-19) are still limited. Nirmatrelvir (PF-07321332), directly targeting the main protease (Mpro) of the severe acute respiratory syndrome coronavirus 2 (SARS-CoV-2), has recently been authorized for treatment of patients with a high risk for progression to severe COVID-19 (*1*, *2*). This oral highly efficient antiviral, which is currently rolled out globally as a first-line treatment, is empowering health care systems to prevent life-threatening cases of COVID-19. In clinical trials in nonhospitalized individuals with mild-to-moderate COVID-19, nirmatrelvir reduced the risk of hospitalization or death by 89% (*2*, *3*). This is superior to the 31% reduction reported for the only other oral SARS-CoV-2 inhibitor molnupiravir (*4*), a nucleoside analog that induces mutations in the viral RNA (vRNA) genome, approved for treatment of individuals at risk for progression to severe COVID-19 who lack alternative treatment options (*5*). The efficacy of nirmatrelvir was comparable to that of the parenteral polymerase inhibitor remdesivir, reported to reduce hospitalization or death by 87% (*6*), approved for patients with severe COVID-19 or an increased risk for progression to severe COVID-19 (*7*–*9*). The role of parenterally administered monoclonal antibodies for COVID-19 treatment is more complex, as their efficacy varies greatly depending on the circulating variant. Thus, omicron variants are resistant to several antibodies with efficacy against previously circulating variants (*10*). Until very recently, bebtelovimab remained the only approved monoclonal antibody targeting SARS-CoV-2 spike protein with proven activity against all circulating variants (*11*). Last, nirmatrelvir is being considered for treatment of long-term negative health effects of SARS-CoV-2 infection, termed long COVID (*12*). However, the high clinical efficacy of nirmatrelvir might be threatened by development of antiviral resistance, as previously observed for virtually all small-molecule inhibitors targeting viruses such as influenza, hepatitis B and C, and human immune deficiency virus (*13*), in particular, when given as monotherapy.

To facilitate population monitoring for potentially emerging nirmatrelvir-resistant SARS-CoV-2 variants, we aimed to identify nirmatrelvir resistance–associated substitutions. To predict the likelihood of resistance selection in treated individuals, we further aimed to evaluate fitness of nirmatrelvir-resistant variants. Moreover, we investigated sensitivity of these variants to remdesivir and bebtelovimab and evaluated prospects for combination treatment with nirmatrelvir and these compounds.

## RESULTS AND DISCUSSION

**Fig. 1.**
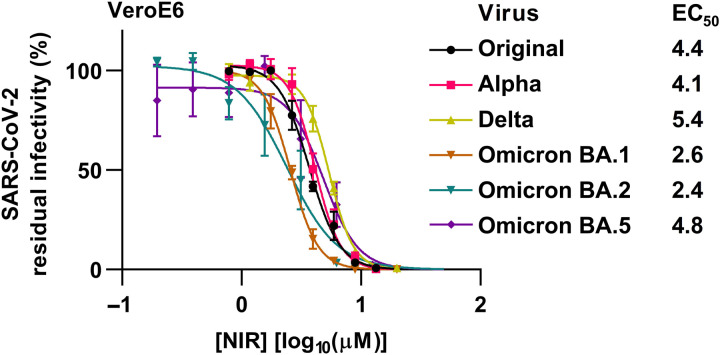
Nirmatrelvir had similar efficacy against the original SARS-CoV-2 virus and the alpha, delta, and omicron BA.1, BA.2, and BA.5 variants. Short-term concentration-response treatments of the original SARS-CoV-2 virus, as well as the alpha, delta, and specified omicron variants with nirmatrelvir (NIR) were carried out in VeroE6 cells in 96-well plates. Infected cells were visualized by spike protein immunostaining. Data points represent % residual infectivity, calculated by relating the number of infected cells in treated cultures to the mean number of infected cells in 8 or 14 infected nontreated cultures and are means of four or seven replicates with SEM. Curves and EC_50_ were determined in GraphPad Prism 8.0.0.

We confirmed the high efficacy of nirmatrelvir against original SARS-CoV-2 in VeroE6 monkey kidney and A549-hACE2 human lung cells (*14*, *15*), recording median effective concentrations (EC_50_) of 4.4 and 0.08 μM, respectively (fig. S1). The observed cell line–specific differences in EC_50_ are explained by differences in the expression level of efflux transporter P-glycoprotein (*16*). Moreover, we demonstrated a similar efficacy of nirmatrelvir against epidemiologically relevant variants, including alpha, delta, and omicron BA.1, BA.2, and BA.5 (Fig. 1). We further confirmed the low cytotoxicity of nirmatrelvir in these cell lines (fig. S2).

**Fig. 2.**
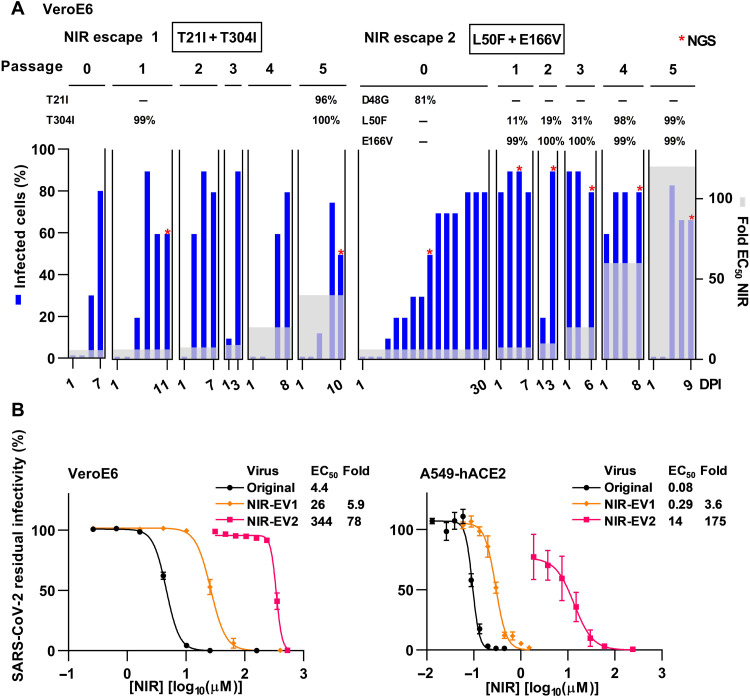
Selection of SARS-CoV-2 nirmatrelvir escape variants in an infectious cell culture system. (**A**) Induction of SARS-CoV-2 escape from nirmatrelvir (NIR) by serial viral passage under increasing drug pressure. Two escape experiments, each comprising a primary escape culture (passage 0) and five passage cultures (passages 1 to 5), were done. Key substitutions acquired in Mpro are specified in boxes on top of the graphs. Frequency of acquired Mpro substitutions is indicated for the respective passages (compare table S1). −, specified substitution not detected in ≥10% of genomes; no entry, passage not analyzed; *, time points analyzed by next-generation sequencing (NGS). Left *y* axes: % SARS-CoV-2–infected culture cells determined by spike protein immunostaining; right *y* axes: fold-EC_50_ nirmatrelvir applied. *X* axes: day post-infection (DPI) for each culture. (**B**) Short-term concentration-response treatments of specified nirmatrelvir escape viruses (NIR-EV1 and NIR-EV2) in VeroE6 or A549-hACE2 cells with nirmatrelvir (NIR). Infected cells were visualized by spike protein immunostaining. Data points represent % residual infectivity, calculated by relating the number of infected cells in treated cultures to the mean number of infected cells in 8 or 14 infected nontreated cultures and are means of 4 or 7 replicates with SEM. Curves and EC_50_ were determined in GraphPad Prism 8.0.0. Fold resistance (Fold) was determined as EC_50variant_/EC_50original virus_ using rounded values.

To identify SARS-CoV-2 nirmatrelvir resistance substitutions, we induced SARS-CoV-2 escape from nirmatrelvir in VeroE6 cells by serial viral passage under increasing drug concentrations. In control cultures, original SARS-CoV-2 was suppressed by sevenfold EC_50_ nirmatrelvir. In contrast, nirmatrelvir escape viruses from two different escape experiments could overcome up to 40- and 120-fold EC_50_ nirmatrelvir, respectively (Fig. 2A). Higher nirmatrelvir concentrations inhibited spread of escape viruses. Following five viral passages, polyclonal nirmatrelvir escape viruses harbored Mpro substitutions T21I + T304I [polyclonal nirmatrelvir escape virus 1 (NIR-EV1)] or L50F + E166V (NIR-EV2) and showed up to 5.9- or 175-fold resistance to nirmatrelvir, respectively (Fig. 2B and table S1). For the highly resistant NIR-EV2, we studied the dynamics of selection of Mpro substitutions in greater detail: D48G, present in 81% of viral genomes in the primary escape culture, was replaced by E166V, present in 99% of genomes in the first passage culture. The frequency of L50F increased from 11 to 99% between first and fifth passage (Fig. 2A and table S1).

**Fig. 3.**
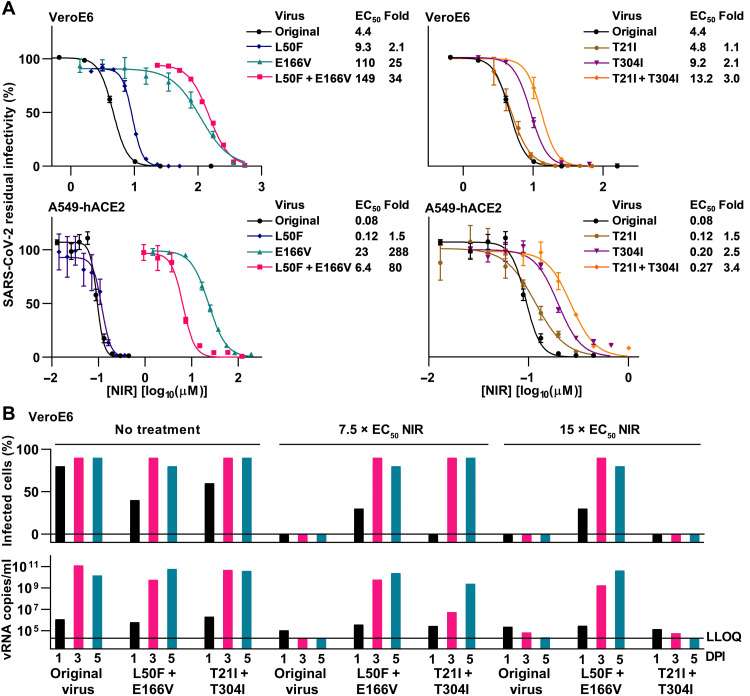
Identification of nirmatrelvir resistance–associated substitutions using reverse genetics. (**A**) Short-term concentration-response treatments of variants with specified engineered resistance-associated substitutions in VeroE6 or A549-hACE2 cells with nirmatrelvir (NIR). For further details, see Fig. 2B. (**B**) Longer-term treatments of variants with specified engineered resistance-associated substitutions in VeroE6 cells with the specified fold-EC_50_ nirmatrelvir (NIR). On the specified DPI, the % SARS-CoV-2–infected culture cells were determined by spike protein immunostaining, and viral RNA (vRNA) titers in culture supernatants were determined by RT-qPCR. LLOQ, lower limit of quantification of the RT-qPCR assay.

We confirmed the significance of the identified putative resistance substitutions by reverse genetics using a bacterial artificial chromosome (BAC) clone corresponding to the original SARS-CoV-2 variant used for escape experiments (*14*, *17*). In short-term treatments, the L50F + E166V variant showed up to 80-fold nirmatrelvir resistance, with resistance being mainly conferred by E166V; the T21I + T304I variant showed no-to-little resistance (Fig. 3A). In longer-term treatments, both double mutants spread under 7.5-fold EC_50_ nirmatrelvir, a concentration with the capacity to suppress the original virus. However, only the L50F + E166V variant could spread under 15-fold EC_50_ nirmatrelvir (Fig. 3B).

A comparison of nirmatrelvir concentrations in cell culture and in humans suggested that the identified substitutions are likely to confer clinically relevant resistance. In clinical trials, nirmatrelvir maximum serum concentrations (*C*_max_) of 4.4 μM were reported (*2*). Thus, *C*_max_/EC_50_ ratios of 1 and 55 for the original SARS-CoV-2 were reduced to 0.03 and 0.7 for the most resistant L50F + E166V variant and to 0.01 and 0.3 for the most resistant polyclonal escape virus in VeroE6 and A549-hACE2 cells, respectively.

**Fig. 4.**
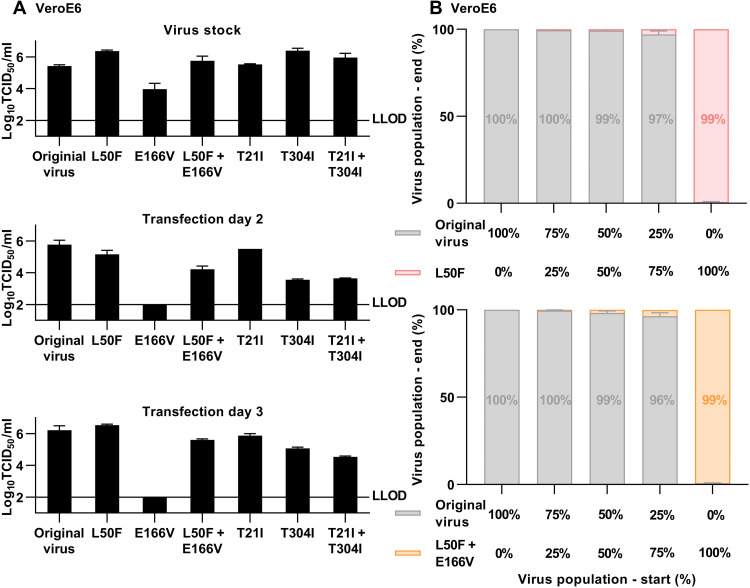
Identified SARS-CoV-2–resistant variants had high fitness but did not outcompete the original virus in an infectious system. (**A**) Viral infectivity titers in virus stocks generated from supernatants of viral passage cultures or of supernatants derived from transfected cultures for SARS-CoV-2 variants with specified engineered resistance-associated substitutions in VeroE6 cells. Data points represent 50% tissue culture infectious doses (TCID_50_)/ml and are means of four replicates with SEM. LLOD, lower limit of detection. (**B**) Competition experiments with simultaneous infection with the original virus and the L50F variant (top) or the original virus and the L50F + E166V variant (bottom) in VeroE6 cells. *X* axes: Percentage of the specified virus populations in the inoculum used for infection at the start of the experiment. *Y* axes: Percentage of virus populations at the end of the experiment, following viral spread to most culture cells, as determined by NGS; virus populations are color-coded.

**Fig. 5.**
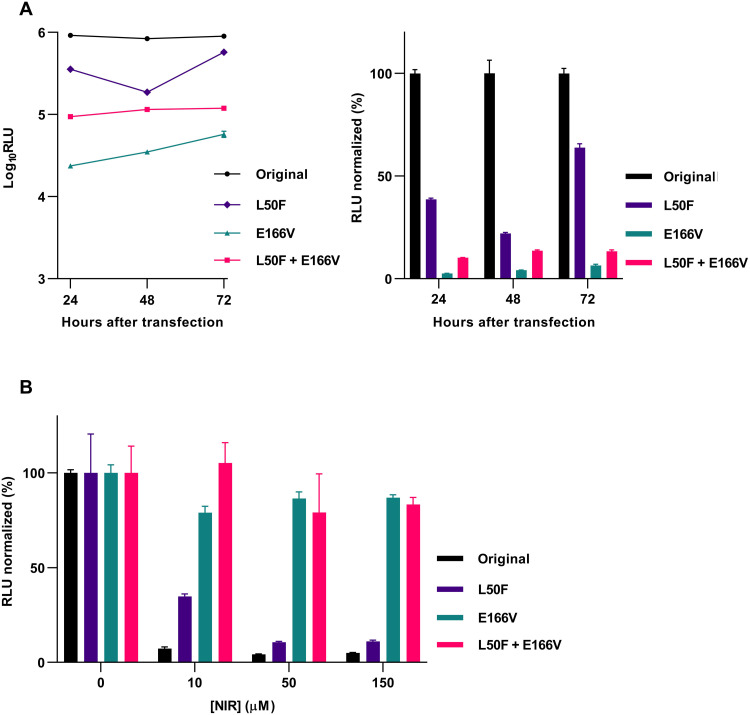
Effect of the identified nirmatrelvir resistance–associated substitutions on SARS-CoV-2 replication. Data are based on measurements of luciferase activity of replicons with the specified substitutions following transfection of VeroE6 cells. (**A**) Left: Data points represent relative light units (RLU) determined at the specified time points after transfection and are means of six replicates with SEM. Right: Data points represent % luciferase activity at the specified time points after transfection relative to the mean luciferase activity of the original virus and are means of six replicates with SEM. (**B**) Data points represent % luciferase activity under treatment with the specified concentrations of nirmatrelvir (NIR) relative to the mean luciferase activity of the respective nontreated controls and are given as means of six replicates with SEM.

**Fig. 6.**
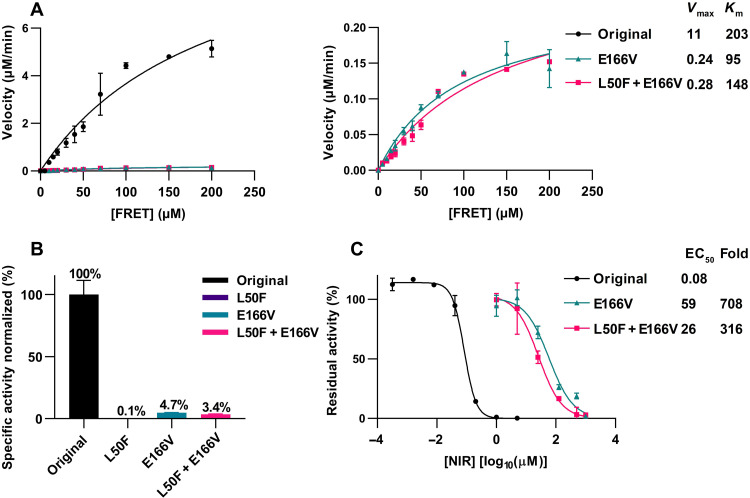
Effect of the identified nirmatrelvir resistance–associated substitutions on the activity of Mpro. (**A**) Enzyme kinetics of Mpro variants measured using an nsp4-nsp5 substrate peptide labeled with a fluorescence quencher pair Dabcyl-Edans (FRET) and excitation and emission wavelengths of 360 nm and 505 nm, respectively. Data points represent initial velocities at varying substrate concentrations. Results are given as means of two replicates with SEM. Initial velocities were plotted as Michaelis-Menten curves, and *V*_max_ and *K*_m_ were determined in GraphPad Prism 8.0.0. *V*_max_ and *K*_m_ values are given in μM/min and μM, respectively. Left: Data points derived from kinetic experiments with the original Mpro and the E166V and L50F + E166V variants. Right: Data points derived from kinetic experiments with the E166V and L50F + E166V variants plotted on a different *y* axis. A Michaelis-Menten curve could not be determined for the L50F variant due to its low enzymatic activity at the conditions used in this assay [see (B)]. (**B**) Data points represent % specific activities at a substrate concentration of 50 μM relative to the mean specific activity of the original Mpro. Results are given as means of two replicates with SEM. (**C**) Data points represent % residual activity of Mpro variants under treatment with varying concentrations of nirmatrelvir (NIR) relative to the mean activity of the respective nontreated controls. Results are given as means of two replicates with SEM. Curves and EC_50_ values are determined in GraphPad Prism 8.0.0. Fold resistance (Fold) was determined as EC_50variant_/EC_50original_ Mpro using non-rounded values. A treatment curve could not be determined for the L50F variant due to its low enzymatic activity [see (B)].

The engineered double mutants showed high fitness in the infectious cell culture system, as their infectivity titers were comparable to those of the original virus in transfection/passage cultures several days after transfection/infection (Fig. 4A). Further, their Mpro was genetically stable, as it had not acquired additional substitutions following four viral passages (table S2). In these assays, L50F had no apparent fitness cost and compensated for fitness cost of E166V, as it rescued low infectivity of the E166V variant and as the E166V variant had acquired L50F in Mpro following four viral passages (Fig. 4A and table S2). In direct competition experiments in this infectious system, the original virus showed higher fitness than the L50F and L50F + E166V variants (Fig. 4B). In agreement with these data, L50F, E166V, and L50F + 166V all decreased replication of a SARS-CoV-2 subgenomic replicon corresponding to the variant used for escape experiments (Fig. 5A) (*17*). E166V conferred the highest and L50F the lowest decrease in replication, and L50F partially rescued replication impairment conferred by E166V. In line with the replicon data, L50F, E166V, and L50F + E166V all decreased Mpro activity (Fig. 6, A and B). In contrast to the replicon data, L50F decreased Mpro activity more than E166V. This discrepancy might be explained by E166V having a stronger effect than L50F on other Mpro functions such as cleavage at alternative junctions of the viral polyprotein or interaction with other viral or cellular proteins during replication. Thus, in stringent head-to-head comparisons in three different in vitro systems, L50F, E166V, and L50F + E166V conferred a fitness cost. L50F compensated for fitness cost of the main resistance substitution E166V in infectious and replicon systems. In an infectious system, mimicking the complete viral life cycle including viral spread, the L50F and L50F + E166V variants showed remarkably high fitness. This high fitness might favor selection and spread of resistant variants. However, our competition data suggest that these variants might not become dominant in human populations in the absence of nirmatrelvir selection pressure. Moreover, in replicon and Mpro assays, we confirmed the specific contributions of E166V and L50F + E166V to nirmatrelvir resistance (Figs. 5B and 6C).

**Fig. 7.**
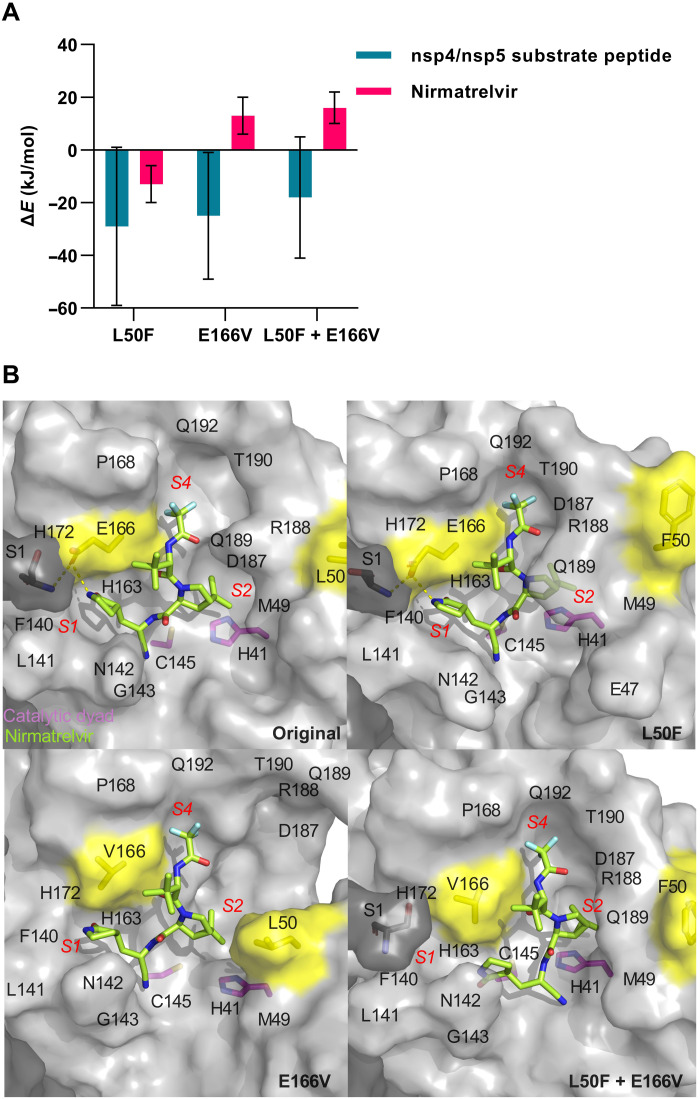
Identified SARS-CoV-2 resistance-associated substitutions impaired molecular Mpro-nirmatrelvir interactions. (**A**) Interaction energy differences (Δ*E*_variant-original_) for binding of nirmatrelvir or of the nsp4/nsp5 substrate peptide to Mpro with L50F, E166V, or E166V + L50F compared to original Mpro, as determined from MDS. (**B**) Final Mpro-nirmatrelvir conformations extracted from MDS with labeling of subsites *S1*, *S2*, and *S4* (red) and subsite residues (black). The important interactions of E166 in monomer A (light gray) with S1 in monomer B (dark gray) and with nirmatrelvir (green) are indicated by yellow dashes. The catalytic dyad (H41 and C145) is shown in magenta, and L50F and E166V are shown in yellow. Noncarbon atoms are colored as follows: N, blue; O, red; F, light blue.

To understand the effects of the identified substitutions on a molecular level, we carried out molecular dynamics simulations (MDS) on Mpro-nirmatrelvir and Mpro–substrate peptide complexes (Supplementary Text and fig. S3). Analysis of the backbone atoms’ root mean square displacements showed that the protein structure was stable throughout the simulations on the complex with nirmatrelvir and the complex with peptide substrate (figs. S4 and S5, respectively). The MDS suggested that E166V and L50F + E166V weakened and that L50F improved nirmatrelvir binding (Fig. 7A and table S3). The fact that E166V weakened binding of Mpro to nirmatrelvir could be explained by the loss of the enzymatically important nirmatrelvir-E166-S1 interactions (*18*), and the loss of nirmatrelvir interactions with Mpro residues 187 to 192 leading to an opening of subsites *S2* and *S4* (Fig. 7B and figs. S6 and S7). L50F likely improved Mpro-nirmatrelvir binding by a gain of nirmatrelvir interactions with R188 and T190 at subsite *S4* (Fig. 7B and figs. S6 and S7). In the double mutant, in addition to this gain of interactions, a reduction of nirmatrelvir interactions with S1, F140, L141, H163, and H172 in subsite *S1* was observed (Fig. 7B and figs. S6 and S7). Consequently, E166V and L50F + E166V might shift the Mpro-nirmatrelvir conformational equilibrium toward noncatalytically competent states, reducing the probability of nirmatrelvir inhibition by 50 and 63%, respectively (fig. S8). The weakened nirmatrelvir binding and reduced inhibition probability could explain the experimentally observed resistance for E166V and L50F + E166V.

In contrast, substrate binding appeared to be improved or unchanged for L50F, E166V, and L50F + E166V (Fig. 7A and table S3), which was in line with previous observations for the single substitutions (*19*). The E166V- and L50F-mediated decreases of Mpro activity (Fig. 6, A and B) could be due to Mpro dimer destabilization and perturbation of the *S1* subsite. In more detail, our MDS suggested that all substitutions led to a disruption of important dimer interactions (S1-F140 and R4-E290; fig. S9), thereby likely destabilizing Mpro dimerization, which is a requirement for catalytic activity (*20*). For E166V and L50F + E166V, our MDS furthermore suggested a disruption of the F140-H163 stacking interaction (fig. S10), which is important for maintaining the *S1* subsite in a catalytically competent conformation (*21*). Our findings are supported by previous studies showing that E166 is of importance for stabilizing the Mpro dimer (*22*) and for stabilizing substrate binding in a catalytically competent conformation through interactions in subsites *S1* and *S4* (*18*). In addition, so far unpublished reverse genetic studies reported that E166A and L50F reduced Mpro dimerization.

**Fig. 8.**
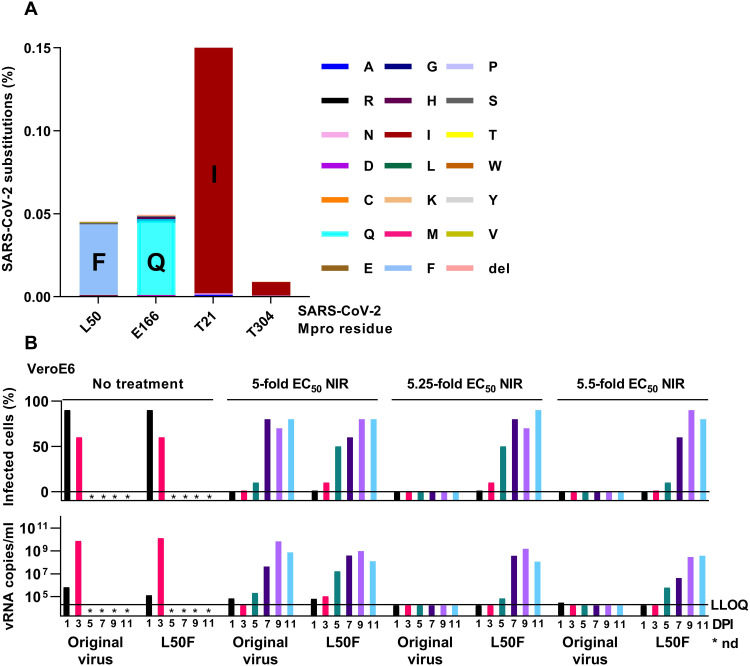
Natural occurrence of resistance-associated substitutions and capacity of preexisting L50F to promote viral escape from nirmatrelvir. (**A**) GISAID database analysis of genetic variation of Mpro sequences. Percentage of viruses with genetic variation at the specified Mpro positions in relation to 10.302.924 analyzed sequences. Specific amino acid residues are color-coded; del, deletion. (**B**) Longer-term treatments of original SARS-CoV-2 virus and the L50F variant in VeroE6 cells with the specified fold-EC_50_ nirmatrelvir (NIR). *nd, not done as culture was stopped because of virus-induced cell death. For further details, see Fig. 3B.

Analysis of sequences derived from the global initiative on sharing all influenza data (GISAID) database prior to widespread use of nirmatrelvir in patients (18 April 2022) revealed that resistance-associated Mpro positions were overall conserved but permitted a certain degree of variation (Fig. 8A and table S4). In particular, we observed a nonnegligible frequency of L50F. To investigate how preexisting L50F influenced nirmatrelvir resistance selection, we carried out head-to-head escape experiments with the original virus and the L50F variant. We found that preexisting L50F promoted nirmatrelvir escape, as the L50F variant escaped from 5.25- and 5.5-fold EC_50_ nirmatrelvir, which suppressed the original virus in this experiment; further, under 5-fold EC_50_ nirmatrelvir, the L50F variant showed accelerated escape kinetics compared to the original virus (Fig. 8B). Following the primary escape (Fig. 8B) and a first viral passage under 10-fold EC_50_ nirmatrelvir, the three escape viruses with preexisting L50F had additionally acquired E166V, S144A, and E166A, respectively (table S5).

As Mpro residues L50 and E166 are highly conserved for SARS-CoV-1 and other sarbecoviruses (fig. S11), our findings are relevant for future outbreaks with emerging sarbecoviruses and contribute to pandemic preparedness.

During revision of this article, Heilmann *et al.* (*23*) reported selection of nirmatrelvir resistance substitutions in a chimeric vesicular stomatitis virus expressing SARS-CoV-2 Mpro in baby hamster kidney-21 (BHK-21) cells. Compared to our study, different substitutions were selected conferring lower resistance, with Q192R + F305L conferring the highest resistance (35-fold). Infectious systems based on full-length SARS-CoV-2 clones, reflecting the complete viral life cycle, appear to be most relevant to model viral resistance and fitness in humans.

In the clinic, rebound of SARS-CoV-2 infection following nirmatrelvir treatment was observed (*24*, *25*), but without association with putative Mpro resistance substitutions. On the other hand, different Mpro substitutions including L50F and E166V were identified in individuals treated with nirmatrelvir, but without an obvious link to a clinically adverse outcome (*2*). In one patient harboring SARS-CoV-2 with Mpro L50F at baseline, E166V was acquired under nirmatrelvir treatment. Additional clinical studies investigating correlates of nirmatrelvir resistance in humans, who experience rebound or breakthrough infections, will be needed to confirm the clinical relevance of the substitution identified in vitro.

**Fig. 9.**
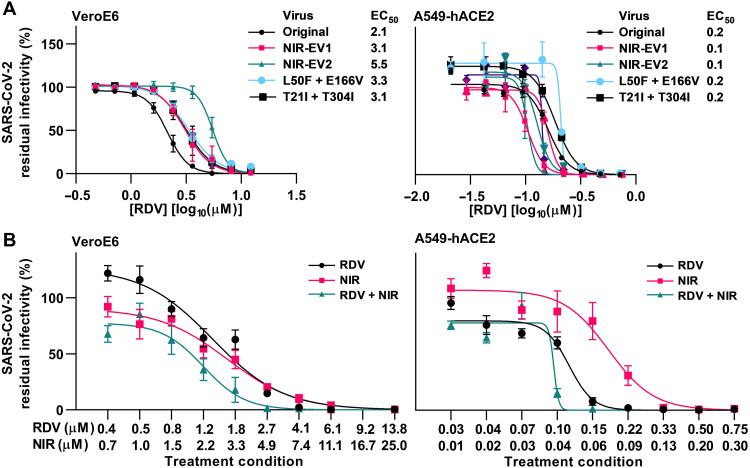
Activity of remdesivir was retained against SARS-CoV-2–resistant variants and enhanced by combination with nirmatrelvir. (**A**) Short-term concentration-response treatments of specified nirmatrelvir escape viruses (NIR-EV1 and NIR-EV2) or variants with specified engineered resistance-associated substitutions in VeroE6 or A549-hACE2 cells with remdesivir (RDV). For further details, see Fig. 2B. (**B**) Short-term concentration-response treatments of original SARS-CoV-2 virus in VeroE6 or A549-hACE2 cells with remdesivir (RDV), nirmatrelvir (NIR), or a combination of both (RDV + NIR). Each of the treatment conditions (indicated by ticks on *x* axis) was defined by the specified concentrations of RDV and NIR, which were applied singly and in combination, resulting in three data points per treatment condition. For further details, see Fig. 2B.

**Fig. 10.**
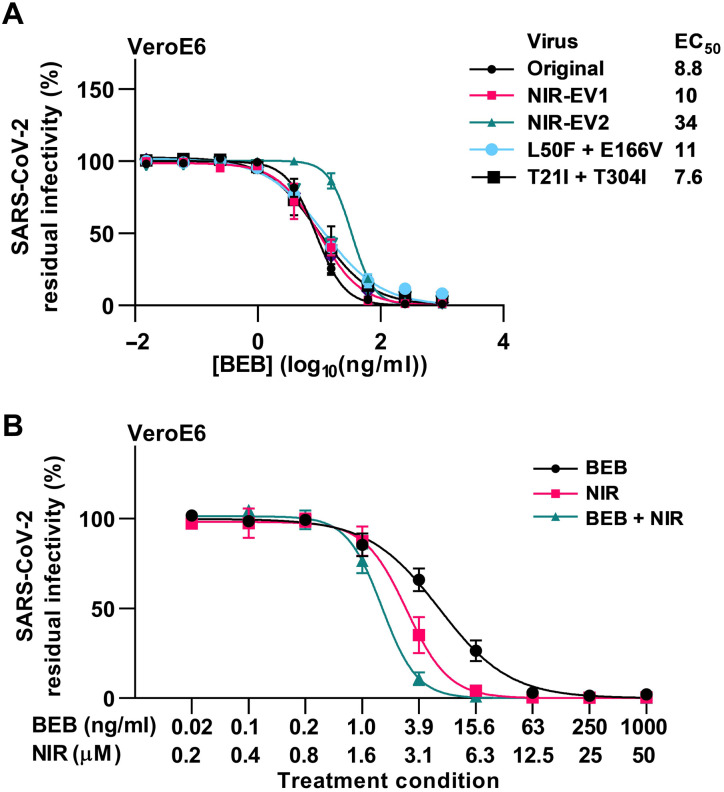
Activity of bebtelovimab was retained against SARS-CoV-2–resistant variants and enhanced by combination with nirmatrelvir. (**A**) Short-term concentration-response treatments of specified nirmatrelvir escape viruses (NIR-EV1 and NIR-EV2) or variants with specified engineered resistance-associated substitutions in VeroE6 cells with bebtelovimab (BEB). For further details, see Fig. 2B. (**B**) Short-term concentration-response treatments of original SARS-CoV-2 virus in VeroE6 cells with BEB, nirmatrelvir (NIR), or a combination of both (BEB + NIR). Each of the treatment condition (indicated by ticks on *x* axis) was defined by the specified concentrations of BEB and NIR, which were applied singly and in combination, resulting in three data points per treatment condition. For further details, see Fig. 2B.

Last, we evaluated efficacy of key COVID-19 medications against resistant variants and prospects for combination treatments. We demonstrated that remdesivir showed similar efficacy against nirmatrelvir-resistant variants and the original SARS-CoV-2 (Fig. 9A). Combination treatment with nirmatrelvir and remdesivir showed enhanced efficacy compared to treatment with the individual compounds, with residual infectivity in combination treatments being up to 5.7- and 3.9-fold decreased compared to treatments with nirmatrelvir and remdesivir, respectively (Fig. 9B). In addition, we showed that bebtelovimab retained activity against all nirmatrelvir-resistant variants (Fig. 10A). Combination treatment with nirmatrelvir and bebtelovimab showed enhanced efficacy with an up to 4-fold and 28-fold decrease in residual infectivity compared to treatments with nirmatrelvir and bebtelovimab, respectively (Fig. 10B).

In conclusion, we report the identification of substitutions in SARS-CoV-2 Mpro conferring substantial and likely clinically relevant resistance to the first-in-class oral protease inhibitor nirmatrelvir. Resistance selection and spread in populations might be supported by the relatively high fitness of the resistant variants, a certain flexibility of resistance-associated Mpro residues and suboptimal treatment adherence during home medication. In addition, circulating variants with preexisting L50F could accelerate resistance selection. Sensitivity of nirmatrelvir-resistant variants to the polymerase inhibitor remdesivir and the monoclonal antibody bebtelovimab as well as enhanced efficacy of combination treatments might provide an incentive for evaluation of these combination therapies in clinical trials. Overall, this study provides a basis for monitoring of nirmatrelvir resistance substitutions in populations. As nirmatrelvir showed broad activity against different coronaviruses (*16*), and as the identified resistance-associated Mpro positions were conserved across sarbecoviruses, our findings have implications not only for the current but also for future sarbecovirus outbreaks.

## MATERIALS AND METHODS

### Cell culture

VeroE6 cells (gift from J. Dubuisson) were cultured in Dulbecco’s modified Eagle’s medium (Invitrogen, Paisley, UK) as described (*15*, *26*, *27*). A549-hACE2 cells (InvivoGen, Toulouse, France) were cultured in Dulbecco’s modified Eagle’s medium: Nutrient Mixture F-12 (Gibco, Paisley, UK) as described (*15*, *26*, *27*). Both culture media were supplemented with 10% heat-inactivated fetal bovine serum (Sigma-Aldrich, St. Louis, MO, USA), penicillin (100 U/ml), and streptomycin (100 μg/ml; Gibco/Invitrogen Corporation, Carlsbad, CA, USA). A549-hACE2 culture medium was in addition supplemented with puromycin (0.5 μg/ml; InvivoGen, Toulouse, France). Cells were maintained at 37°C and 5% CO_2_ and subcultured every 2 to 3 days.

### SARS-CoV-2 virus stocks

All virus stocks were generated in VeroE6 cells, and sequence was confirmed by next-generation sequencing (NGS) in relation to the patient isolate or BAC clone they originated from as described in the “Sequencing analysis of SARS-CoV-2 genomes” section.

#### SARS-CoV-2 original virus: SARS-CoV-2 D614G

The second viral passage stock with an infectivity titer of 5.5 log_10_ 50% tissue culture infectious doses (TCID_50_)/ml was based on the isolate SARS-CoV-2/human/Denmark/DK-AHH1/2020 (*14*) (GenBank: MZ049597), with a spike protein sequence as reported in early outbreak isolates such as the Wuhan-Hu-1 isolate with the D614G mutation. This stock was used for induction of viral escape and as a reference in short-term concentration-response treatments, longer-term treatments, and viral fitness competition experiments.

#### SARS-CoV-2 alpha variant

The second viral passage stock with an infectivity titer of 6.7 log_10_ TCID_50_/ml was based on the isolate SARS-CoV-2/human/DNK/DK-AHH1/2021 (GenBank: OK041529) (*28*). This stock was used for short-term concentration-response treatments.

#### SARS-CoV-2 delta variant

The second viral passage stock with an infectivity titer of 6.0 log_10_ TCID_50_/ml was based on the isolate SARS-CoV-2/human/DNK/DK-AHH3/2021 (GenBank: OP271297). This stock was used for short-term concentration-response treatments.

#### SARS-CoV-2 omicron BA.1 variant

The second viral passage stock with an infectivity titer of 5.5 log_10_ TCID_50_/ml was based on the isolate SARS-CoV-2/human/DNK/DK-AHH4/2021 (GenBank: OP271296). This stock was used for short-term concentration-response treatments.

#### SARS-CoV-2 omicron BA.2 variant

The second viral passage stock with an infectivity titer of 6.5 log_10_ TCID_50_/ml was based on the isolate SARS-CoV-2/human/DNK/DK-AHH5/2022 (GenBank: OP722493). This stock was used for short-term concentration-response treatments.

#### SARS-CoV-2 omicron BA.5 variant

The second viral passage stock with an infectivity titer of 6.5 log_10_ TCID_50_/ml was based on the isolate SARS-CoV-2/human/DNK/DK-AHH6/2022 (GenBank: OP722492). This stock was used for short-term concentration-response treatments.

#### Other virus stocks

Polyclonal escape virus stocks were generated and used as described in the “Induction of SARS-CoV-2 escape” section. Stocks of SARS-CoV-2 recombinants were generated and used as described in the next section.

### SARS-CoV-2 recombinants and transfections

Recombinants were based on the BAC clone encoding the sequence of the SARS-CoV-2 D614G isolate SARS-CoV-2/human/Denmark/DK-AHH1/2020 (*17*). For generation of variants, point mutations were engineered by in-fusion and mega-primer–based cloning using sequence-specific primers.

In vitro RNA transcripts of the SARS-CoV-2 genome were generated as described (*17*). In brief, Not I–linearized plasmids were purified using the Zymo DNA Clean & Concentrator-25 Kit with Zymo-Spin IC-XL columns (ZR BAC DNA Miniprep Kit, ZymoResearch, Irvine, CA, USA) and in vitro transcribed with the mMESSAGE mMACHINE T7 Transcription Kit (Thermo Fisher Scientific, Waltham, MA, USA). Transfection of 200,000 VeroE6 cells per well plated the previous day in 12-well plates (Thermo Fisher Scientific, Roskilde, Denmark) was carried out using 2 μg of in vitro RNA transcripts, quantified with the Qubit RNA BR Assay Kit (Thermo Fisher Scientific, Waltham, MA, USA) and Lipofectamine 2000 (Thermo Fisher Scientific) in Opti-MEM I (Invitrogen, Waltham, MA, USA); as an exception, for the L50F, 1 μg of RNA transcripts was used. Cell culture supernatants were collected on selected days after transfection and stored at −80°C. Transfection efficacy was evaluated by inspection of transfection cultures for cytopathogenic effects (CPEs) in an inverted light microscope and by determination of supernatant SARS-CoV-2 infectivity titers as described in the “Determination of SARS-CoV-2 infectivity titers” section.

The first viral passage virus stocks were generated by inoculation of VeroE6 cells, plated the previous day at 3 million cells per T80 flask (Thermo Fisher Scientific, Roskilde, Denmark), with 250 μl of supernatant derived from transfection cultures at day 2 or 3 after transfection. As an exception, for the L50F variant, a second passage was done for virus stock generation. From infected first- or second-passage cultures, cell culture supernatants were collected at the peak of infection, subjected to NGS for sequence confirmation, and stored at −80°C. Virus stocks were used for short-term concentration-response treatments, longer-term treatments, and viral fitness competition experiments.

### Serial passage of engineered SARS-CoV-2 recombinants

To investigate their genetic stability, engineered SARS-CoV-2 recombinants were subjected to four viral passages. VeroE6 cells, plated the previous day at 1 million cells per T25 flask (Thermo Fisher Scientific, Roskilde, Denmark), were inoculated with 250 μl of supernatant derived at the peak of infection from the preceding culture. Supernatant derived at the peak of infection from the fourth passage culture was subjected to NGS.

### Inhibitors/monoclonal antibody for SARS-CoV-2 treatments

All small-molecule inhibitors were purchased from Acme Bioscience (Palo Alto, CA, USA), dissolved in dimethyl sulfoxide (Sigma-Aldrich, St. Louis, MO, USA), and stored at −20°C. Bebtelovimab was purchased from Cell Sciences (#CPC539B, Newburyport, MA, USA) and stored at −80°C.

### Cell viability assays

To evaluate cytotoxic effects of the studied inhibitors, cell viability assays were done as described (*15*, *26*, *27*) using the CellTiter 96 AQueous One Solution Cell Proliferation Assay (Promega, Madison, WI, USA). VeroE6 or A549-hACE2 cells, plated the previous day at 10,000 cells per well in 96-well flat-bottom plates (Thermo Fisher Scientific, Roskilde, Denmark), were treated with the specified concentrations of inhibitors, including three replicate wells per concentration and 10 nontreated control wells. Following incubation for 46 to 50 hours at 37°C and 5% CO_2_, CellTiter 96 AQueous One Solution Reagent was added at 20 μl per well followed by incubation for 1.5 to 2 hours at 37°C and 5% CO_2_ and determination of absorbance at 492 nm using a FLUOstar OPTIMA 96-well plate reader (BMG LABTECH, Offenburg, Germany). For calculation of cell viability in %, absorbance values from individual treated wells were related to the mean absorbance of nontreated control wells. Data points are means of triplicates with SEMs. Sigmoidal concentration-response curves were fitted, and 50% cytotoxic concentration values were calculated using GraphPad Prism 8.0.0 with a bottom constraint of 0 applying the equation *Y* = Top/[1 + 10^(log10EC50 − *X*)*HillSlope^].

### Induction of SARS-CoV-2 escape

VeroE6 cells, plated the previous day at 1 million cells per T25 flask, were inoculated at 0.00002 multiplicity of infection (MOI) with the SARS-CoV-2 D614G stock and treated with nirmatrelvir at concentrations selected to suppress, but not eradicate, viral infection. Every 2 to 3 days, cell culture supernatants were collected and stored at −80°C for NGS, cells were subcultured as required, aiming to maintain semiconfluent cell layers, and fresh supernatant containing inhibitors at specified concentrations was added. Upon subculturing, replicate cultures were plated on chamber slides for immunostainings to determine the percentage of SARS-CoV-2–infected culture cells as described in the next section. Results from immunostainings were used to adapt inhibitor concentrations. Two nirmatrelvir escape experiments were carried out, each comprising a primary escape culture followed by five passages under increasing inhibitor concentrations, further detailed in table S1.

NIR-EV1 and NIR-EV2 stocks were generated by inoculation of VeroE6 cells, plated at 3 million cells the previous day in T80 flasks, with 15 μl of supernatant derived from passage 5 day 10 from escape 1 or passage 5 day 9 from escape 2. From these inoculated cultures, supernatants were collected at the peak of infection, stored at −80°C, and subjected to NGS. Virus stocks were used for short-term concentration-response treatments and longer-term treatments.

### SARS-CoV-2 spike protein immunostaining

Immunostaining was used for monitoring of SARS-CoV-2–infected VeroE6 cell cultures for induction of viral escape and longer-term treatments as described (*15*, *27*). In these cultures, following cell splitting, replicate cultures were plated in eight-well chamber slides (Thermo Fisher Scientific, Rochester, NY, USA). The next day, slides were submerged in methanol (J.T.Baker, Gliwice, Poland) for 20 min to fix cells and inactivate SARS-CoV-2. Slides were washed three times with phosphate-buffered saline (PBS; Sigma-Aldrich, Gillingham, UK) with 0.1% Tween 20 (Sigma-Aldrich, St. Louis, MO) (PBS-tween) and incubated with first antibody SARS-CoV-2 spike chimeric monoclonal antibody (Sino Biological #40150-D004, Beijing, China) diluted 1:1000 in PBS with 1% bovine serum albumin (Roche, Mannheim, Germany) and 0.2% skim milk (Easis, Aarhus, Denmark) (PBSK) for 2 hours. Slides were washed two times with PBS-tween and incubated with second antibody Alexa Fluor 488 goat anti-human immunoglobulin G (IgG) (H+L) (Invitrogen #A-11013, Paisley, UK) diluted 1:500 and Hoechst 33342 (Invitrogen, Paisley, UK) diluted 1:1000 in PBS-tween for 20 min. Percentages of SARS-CoV-2 spike protein–positive cells were evaluated by fluorescence microscopy (ZEISS Axio Vert.A1, Jena, Germany), using the following designations: 0% infected cells (no cells infected), single infected cells, and 10 to 90% infected cells (in steps of 10%).

### Short-term concentration-response treatments

VeroE6 or A549-hACE2 cells, plated the previous day at 10,000 cells per well in 96-well flat-bottom plates (Thermo Fischer Scientific, Roskilde, Denmark), were inoculated with the specified SARS-CoV-2 virus stocks as described (*15*, *26*, *27*); the size of the inoculum was determined in pilot assays aiming at 800 to 3000 infected cells in infected nontreated wells and the absence of CPE at the end of the experiment. Cultures were incubated for 1 hour at 37°C and 5% CO_2_ and treated with specified concentrations of inhibitors as described (*15*) with four or seven replicates per concentration; 8 or 14 infected and nontreated and 8 or 12 noninfected and nontreated replicate wells were included in each assay.

Following incubation for 46 to 50 hours at 37°C and 5% CO_2_, plates were subjected to SARS-CoV-2 spike protein immunostaining. Plates were submerged in methanol for 20 min and washed three times with PBS-tween, followed by blocking of endogenous peroxidase activity with H_2_O_2_ for 10 min, two washes with PBS-tween, and blocking by PBSK for 30 min. Plates were incubated with first antibody SARS-CoV-2 spike chimeric monoclonal antibody (Sino Biological #40150-D004, Beijing, China) diluted 1:5000 in PBSK for 2 hours, washed two times with PBS-tween, and incubated for 1 hour with second antibody F(ab′)2-Goat anti-human IgG Fc Cross-Adsorbed Secondary Antibody, HRP (Invitrogen, #A24476, Carlsbad, CA, USA) or Goat F(ab′)2 Anti-Human IgG – Fc (HRP), preadsorbed (Abcam, ab#98595, Cambridge, UK), diluted 1:2000 in PBSK. Following two washes with PBS-tween, cells were stained with the DAB substrate BrightDAB Kit (Immunologic # BS04-110, Duiven, The Netherlands).

The number of single SARS-CoV-2 spike protein–positive cells per well was evaluated using the ImmunoSpot Series 5 UV Analyzer (CTL Europe GmbH, Bonn, Germany). Representative images from concentration-response antiviral treatment assays are shown by Zhou *et al.* (*26*). Mean counts of noninfected nontreated wells, which were usually <100, were subtracted from counts of individual infected wells. For calculation of % residual infectivity, counts of individual infected treated wells were related to mean counts of infected nontreated wells. Data points are means of four or seven replicates with SEM. Sigmoidal concentration-response curves were fitted, and EC_50_ values were calculated as described previously using GraphPad Prism 8.0.0 with a bottom constraint of 0 applying the equation *Y* = Top/[1 + 10^(log10EC50 − *X*)*HillSlope^]. Fold resistance (Fold) was determined as EC_50variant_/EC_50original virus_. EC_50original virus_ was the mean of several EC_50_ determinations shown in fig. S1. In Figs. 1, 2B, and 3A, a representative treatment curve is shown for the original SARS-CoV-2 virus, and treatment curves included in the same graph were not in all instances carried out in the same experimental setup. However, in each treatment experiment with SARS-CoV-2 variants, the original SARS-CoV-2 virus was included for comparison to ensure reproducibility.

### Longer-term treatments

VeroE6 cells, plated the previous day at 1 million cells per T25 flask, were infected at 0.00002 MOI with the specified SARS-CoV-2 virus stocks as described (*15*). Cells were treated with specified fold EC_50_ of inhibitors upon inoculation and then every 2 days upon subculturing. Upon subculturing, replicate cultures were plated on chamber slides for immunostainings to determine the percentage of SARS-CoV-2–infected cells as described in the “SARS-CoV-2 spike protein immunostaining” section. Further, cell culture supernatants were collected and stored at −80°C to determine vRNA titers by reverse transcription quantitative polymerase chain reaction (RT-qPCR) as described in the “Determination of SARS-CoV-2 RNA titers” section. An infected nontreated culture was included as a positive infection control.

### Combination treatments

Combination of nirmatrelvir with remdesivir or bebtelovimab for inhibition of SARS-CoV-2 was evaluated in short-term treatments in VeroE6 or A549-hACE2 cells as described (*15*). VeroE6 or A549-hACE2 cells, plated the previous day at 10,000 cells per well in 96-well flat-bottom plates, were inoculated with the original SARS-CoV-2 virus stock; the size of the inoculum was determined in pilot assays aiming at 2000 to 4000 infected cells in infected nontreated wells and the absence of CPE at the end of the experiment. Cultures were incubated for 1 hour at 37°C and 5% CO_2_ and treated with specified concentrations of inhibitors/antibody. For all inhibitors/antibodies and their combinations, dilution series were used spanning the inhibitor/antibody EC_50_ values and aiming at residual infectivity values between 0 and 100%. In combination treatments, the same concentrations as in single treatments were used with a fixed ratio. All treatment conditions were evaluated using four or seven replicates including 8 or 14 infected nontreated replicates and 8 or 12 noninfected nontreated replicates. Following incubation for 46 to 50 hours at 37°C and 5% CO_2_, plates were subjected to SARS-CoV-2 spike protein immunostaining, automated counting of single SARS-CoV-2 spike protein–positive cells, and further evaluation as described in the “Short-term concentration-response treatments” section. Fold enhancement of combination treatments compared to treatments with individual compounds was calculated per treatment condition, which was defined by a specific concentration of nirmatrelvir and remdesivir or bebtelovimab, relating % residual infectivities to each other.

### Determination of SARS-CoV-2 RNA titers

The vRNA titers in cell culture supernatants were determined by RT-qPCR as described (*15*). In brief, RNA was extracted using Trizol LS (Life Technologies) and chloroform (Sigma-Aldrich, St. Louis, MO, USA) and purified using the Zymo RNA Clean and Concentrator-5 Kit. For RT-qPCRs, the TaqMan Fast Virus 1-Step Master Mix (Thermo Fisher Scientific) was used with previously described primers and probes (*29*): E_Sarbeco_F (5′-ACAGGTACGTTAATAGTTAATAGCGT-3′), E_Sarbeco_R (5′-ATATTGCAGCAGTACGCACACA-3′), and E_Sarbeco_P (FAM-5′-ACACTAGCCATCCTTACTGCGCTTCG-3′-BHQ1), as well as the LifeCycler 96 System (Roche). The lower limit of quantification of the assay was calculated as (mean of RNA titers in supernatants derived from noninfected control cultures) + (3 SD).

### Determination of SARS-CoV-2 infectivity titers

SARS-CoV-2 infectivity titers in cell culture supernatants from transfection experiments were determined as described previously (*30*). VeroE6 cells, plated the previous day at 10,000 cells per well in 96-well flat-bottom plates, were inoculated with culture supernatants in 10-fold dilution series. Following incubation for 70 to 74 hours at 37°C and 5% CO_2_, plates were subjected to SARS-CoV-2 spike protein immunostaining as described in the “Short-term concentration-response treatments” section. Plates were imaged using the ImmunoSpot Series 5 UV Analyzer (CTL Europe GmbH) and scored infected or noninfected. Infectious titers were calculated as TCID_50_/ml using the Reed-Muench method. The lower limit of detection (LLOD) was 2 log_10_ TCID_50_/ml, defined by the used starting dilution.

### Sequencing analysis of SARS-CoV-2 genomes

SARS-CoV-2 RNA was extracted and purified as described in the “Determination of SARS-CoV-2 RNA titers” section. RT-PCR was used to generate five overlapping amplicons, and the NEBNext Ultra II FS DNA Library Prep Kit (New England BioLabs, Ipswich, MA, USA) was used for library preparations. NGS analysis was done as described (*14*, *17*, *27*, *30*).

### Viral fitness competition experiment

VeroE6 cells, plated the previous day at 1 million cells per T25 flask, were infected simultaneously with original SARS-CoV-2 and the L50F variant or with original SARS-CoV-2 and the L50F + E166V variant (*15*). Control cultures infected with only one of these viruses were included. Infections were done at a total MOI of 0.0001, while the % of each virus in the inoculum used for infection at the start of the experiment varied as specified. Each condition was tested in three replicate cultures. Each day, cell culture supernatant was harvested and stored at −80°C, cells were washed once with PBS, and fresh medium was added. Using supernatants derived on day 4 post-infection, when most culture cells were estimated to be infected by evaluation of CPE, the frequencies (%) of the respective virus populations were determined by NGS based on the frequency of L50F. Frequencies are means of measurements from the three replicate cultures.

### SARS-CoV-2 replicon assays

The nLuc ΔS-E-M replicon, generation of RNA in vitro transcripts, transfection conditions, and determination of luciferase activity were previously described (*17*). In brief, VeroE6 cells, plated the previous day at 100,000 cells per well in 24-well plates, were transfected with 500 ng of RNA transcripts for 1 hour. Then, the transfection reaction was replaced by fresh medium with or without nirmatrelvir at the specified concentrations. The luciferase reporter activity was measured in lysates of transfected cell cultures using the Nano-Glo Luciferase Assay System (Promega, Madison, WI, USA), following the manufacturer’s guidelines. For each condition, duplicate cultures were carried out, and for each culture, measurements were carried out in three technical replicates. For each experiment, a mock transfection with no RNA input was carried out. Measurements were recorded using the Synergy LX Multi-Mode Microplate Reader (BioTek, Winooski, VT, USA) as relative light units (RLU). For each experiment and for each condition, a baseline measurement was carried out 1 hour after transfection. Baseline measurements were subtracted from the subsequent measurements at 24, 48, and 72 hours after transfection. In addition, for each plate recording, empty wells were included serving as blank controls. For each plate, the mean of blank controls was subtracted from all experimental measurements. For transfected nontreated cultures, RLU were normalized by relating to the mean RLU of the original SARS-CoV-2 at each time point and given in %. For transfected and treated cultures, RLU determined 24 hours after transfection and treatment initiation were normalized by relating to the mean RLU of the corresponding nontreated culture and given in %.

### Mpro enzymatic activity assays

The SARS-CoV-2 Mpro variants were purchased from KactusBio (Cambridge, MA, USA). The enzyme activities were measured using the fluorescence resonance energy transfer (FRET) substrate Dabcyl-KTSAVLQ/SGFRKM-E(Edans)-NH2, which was purchased from BioPeptide (San Diego, CA, USA). For the Edans standard curve, *K*_m_ and *V*_max_ measurements, as well as the EC_50_ determinations, a 20 mM Hepes, adjusted to pH 6.5 using NaOH, 120 mM NaCl, 0.4 mM EDTA, 4 mM dithiothreitol, and 20% glycerol buffer, was used (*31*). All fluorescence measurements were carried out on FLUOstar OPTIMA (BMG LabTech, Germany) in black 96-well flat-bottom microplates (Greiner Bio-One, Germany) with a working volume of 100 μl. The fluorescence was measured with filters for excitation at 360 nm and emission at 505 nm. Reactions were monitored every 30 s for an hour, and the initial velocities (unit: μM/min) were determined from the initial linear region of the product versus reaction time graph corresponding to less than 10% substrate conversion. For the Edans standard curve, 200 nM original Mpro was mixed with FRET substrate to achieve the specified final concentrations in the range of 0 to 100 μM. The reactions were monitored every 90 s for an hour. During the reactions, the fluorescence signal reached a plateau, indicating that all substrates were cleaved. The fluorescence signals at the plateaus were plotted as relative fluorescence units against the initial concentration of FRET substrate, and linear regression was performed to generate a standard curve (fig. S12). The *K*_m_ and *V*_max_ measurements were carried out using 100 nM Mpro for the original Mpro and for the E166V and L50F + E166V variants. The Mpro variants were mixed with FRET substrate to achieve the specified final concentrations in the range of 0 to 200 μM. The initial velocities in μM/min were plotted against the initial FRET concentration in GraphPad Prism 8.0.0 to determine *K*_m_ and *V*_max_ using the Michaelis-Menten equation $V_{0}=\frac{V_{\max}\cdot {\left[S\right]}_{0}}{K_{m}+{\left[S\right]}_{0}}.$

The specific activities of the Mpro variants were determined using 100 nM Mpro for the original Mpro and for the E166V and L50F + E166V variants, while 300 nM Mpro was used for the L50F variant. The Mpro variants were mixed with 50 μM FRET substrate. The initial velocities were converted to specific activities (unit: μmolmin^−1^g^−1^) using the following equation: $\text{Specific activity}=\frac{\text{Initial velocity}\cdot Volume}{{Mass}_{protein}}$. The reaction volume was 100 μl, and the variants had a molecular weight of 33.8 kDa.

For the EC_50_ calculations, the Mpro variants were incubated with varying concentrations of nirmatrelvir for 30 min at room temperature. The reactions were initiated by adding 20 μl of substrate to reach a final concentration of 100 nM Mpro and 40 μM FRET substrate. The initial velocities were normalized with respect to the initial velocity when no nirmatrelvir incubations were performed for each Mpro variant and plotted against the final nirmatrelvir concentration to determine EC_50_ using GraphPad Prism 8.0.0 as described in the “Short-term concentration-response treatments” section.

### Molecular dynamics simulations

#### Preparation of systems

Coordinates of SARS-CoV-2 Mpro with nirmatrelvir [Protein Data Bank (PDB) entry: 7vh8 (*32*)] and nsp4/nsp5 substrate peptide [PDB entry: 7mgs (*33*)] bound, respectively, were obtained from the PDB (fig. S3). PyMOL version 2.5.0 was used for preparing the systems for the simulations: building missing residues, deleting ions and ligands (other than nirmatrelvir), applying symmetry operations to form the Mpro dimer, and introducing substitutions (L50F, E166V, or L50F + E166V). Furthermore, the covalent bond between C145 and nirmatrelvir was broken in the Mpro-nirmatrelvir structure, and the intact nirmatrelvir nitrile warhead was built; furthermore, A145 in the Mpro–substrate peptide structure was mutated to C145 (fig. S13). Protonation states for titratable residues at pH 7.4 were assigned on the basis of p*K*_a_ (where *K*_a_ is the acid dissociation constant) calculations using the H++ server (*34*), and histidine protonation site was determined by analysis of the hydrogen bonding network around histidine. The N and C termini of the nsp4/nsp5 substrate peptide were kept neutral to avoid introduction of charges that are not present in the SARS-CoV-2 polyprotein nsp4-nsp5. The Mpro dimers were simulated in water boxes having a dodecahedron shape, and a minimum distance between solute and box edges of 12 Å was applied. Periodic boundary conditions were applied in all three Cartesian directions. Sodium and chloride ions were added to a concentration of 0.150 M NaCl corresponding to the cytosolic physiological ionic strength (*35*). The CHARMM36m all-atom protein force field (*36*, *37*) was used in the simulations, and a nirmatrelvir topology file was generated using the CHARMM General Force Field (CGenFF) (*38*, *39*) available through the CGenFF interface (https://cgenff.umaryland.edu).

#### Simulation settings

Simulations on Mpro dimers (original, L50F, E166V, and L50F + E166V) with substrate peptide or nirmatrelvir molecule bound were run in triplicate using Gromacs version 2021.2 (*40*, *41*). The systems were minimized using a steepest descent algorithm until the maximum force was below 1000 kJ mol^−1^ nm^−1^. The simulations were carried out using a leap-frog integrator and an integration time step of 1 fs. After minimization, equilibration was performed in the *NVT* (*N* = constant number of atoms*, V* = constant volume*, T* = constant temperature) ensemble for 100 ps, followed by an equilibration in the *NPT* (*N* = constant number of atoms*, P* = constant pressure*, T* = constant temperature) ensemble for 100 ps. In both steps, position restraints were applied for Mpro–substrate peptide or Mpro-nirmatrelvir, and the temperature coupling was controlled using a modified Berendsen thermostat (*42*) with a time constant of 0.1 ps. For the *NPT* equilibration step, Berendsen pressure coupling (*42*) was used with a time constant of 2.0 ps. The MDS runs were carried out in an *NPT* ensemble at 310 K and 1 bar for 100 ns, of which the first 50 ns were considered equilibration and the last 50 ns were the production run and used in the subsequent analysis. A modified Berendsen thermostat (*42*) was used for temperature coupling with a time constant of 0.1 ps, and the Parinello-Rahman approach (*43*, *44*) was used for pressure coupling with a time constant of 2.0 ps. The Verlet cutoff scheme was used for calculating short-range van der Waals interactions with a cutoff at 12 Å in combination with force switching starting at 10 Å. The particle mesh Ewald method (*45*, *46*) with a 1.6-Å grid spacing was used for calculating long-range electrostatics. Hydrogen bonds were kept rigid using the LINCS algorithm (*47*).

#### Analysis of simulations

Root mean square deviation of atom coordinates was calculated for all Mpro-nirmatrelvir (fig. S4) or Mpro–substrate peptide (fig. S5) atoms relative to the minimized structures using the gmx rms tool in Gromacs. Interaction energies (*E*) between the Mpro dimer and substrate or nirmatrelvir, respectively, were extracted from the simulations using the gmx energy tool in Gromacs. The interaction energies calculated for each of the two substrate/nirmatrelvir molecules bound in the Mpro dimer were averaged, and the values in table S3 and Fig. 7A are averages of the dimer average interaction energies with SEM given as uncertainties. The differences in interaction energy between original and variant Mpro are calculated as follows: Δ*E* = *E*_variant_ − *E*_original_. Thus, negative energy differences indicate improved nirmatrelvir/substrate binding to an Mpro variant, while positive differences indicate weakened binding.

Distance calculations were carried out using VMD version 1.9.4 (*48*). For the inhibition probability plots, distances were calculated between C145 sulfur and the nirmatrelvir cyano carbon, and between G143 amide nitrogen and the nirmatrelvir cyano nitrogen. For the interaction heatmaps, the frequencies of nirmatrelvir-Mpro residues within 3-Å proximity were calculated and converted to percentage of the simulation frames the interactions occurred in. Differences in frequencies (fig. S6) between original and variant Mpro were calculated as follows: Δfrequency(interactions) = frequency(interactions)_variant_ − frequency(interactions)_original_. Only interactions with differences larger than 5% were included in fig. S6.

### Sarbecovirus Mpro alignment

Sarbecovirus Mpro amino acid sequences were aligned by Geneious Prime 2019.2.3 software. SARS-CoV-2 isolate SARS-CoV-2-WuhanHB (GenBank: NC045512) residues Ser^1^-Gln^306^ were used as the reference. On the basis of the representative sarbecovirus spike receptor-binding domain amino acid sequences, sarbecoviruses phylogenetically cluster into four clades (*49*). Forty-three representative sarbecovirus sequences obtained from GenBank or GISAID from all four clades were chosen for alignment (*49*).

## Supplementary Materials

### This PDF file includes:

Supplementary Text Figs. S1 to S13 Tables S1 to S5 References

## Notes

View/request a protocol for this paper from *Bio-protocol*.
