# Supplementary material for: Nirmatrelvir-resistant SARS-CoV-2 variants with high fitness in an infectious cell culture system
Source: Sci Adv. 2022 Dec 21;8(51):eadd7197. doi: 10.1126/sciadv.add7197 (PMC9770952; doi:10.1126/sciadv.add7197)
Supplement: Supplementary file 1 — Supplementary Text Figs. S1 to S13 Tables S1 to S5 References [file sciadv.add7197_sm.pdf]

Science Advances
AAAS

Supplementary Materials for

Nirmatrelvir-resistant SARS-CoV-2 variants with high fitness in an infectious cell culture system

Yuyong Zhou *et al.*

Corresponding author: Judith Margarete Gottwein, jgottwein@sund.ku.dk

Sci. Adv. **8**, eadd7197 (2022)
DOI: 10.1126/sciadv.add7197

This PDF file includes:

Supplementary Text
Figs. S1 to S13
Tables S1 to S5
References

Supplementary Text: Analysis of main protease (Mpro) molecular dynamics simulations

Mpro structure, function and inhibition

Mpro (nsp5) is one of two cysteine proteases of severe acute respiratory syndrome coronavirus 2 (SARS-CoV-2). The function of Mpro is to cleave the SARS-CoV-2 polyproteins pp1a and pp1ab into mature nonstructural proteins at 11 cleavage sites (*50*). The crystal structure of Mpro with a nsp4/nsp5 peptide bound (*33*) shown in fig. S3a illustrates the nomenclature of the cleavage junction with P5-P1 corresponding to the five C-terminal residues of nsp4 and P1'-P2' corresponding to the two N-terminal residues of nsp5. The cleavage occurs between P1 and P1' (fig. S3b). Nirmatrelvir is a covalent inhibitor of the Mpro, which has been designed to mimic substrate residues P4-P1 (figs. S3c and S3d) (*51*).

Structures of Mpro with substrate peptides bound suggest that peptide bond cleavage is initiated by a nucleophilic attack on the carbonyl carbon (fig. S3b) by the C145 thiolate, which is stabilized by the adjacent H41 (*52*). The attack leads to formation of a high-energy transition state with a negatively charged carbonyl oxygen (*53*), which is stabilized by hydrogen bonds to mainchain amide hydrogens of G143, S144 and C145 forming the oxyanion hole (fig. S13a) (*33*).

For nirmatrelvir, a nucleophilic attack on the nitrile warhead by the C145 thiolate leads to a reversible covalent bond to C145 and thus Mpro inhibition (*32*). Similarly to peptide bond breakage in natural substrates, the nucleophilic attack leads to high-energy transition states with a negatively charged nitrile nitrogen (*54*), which is stabilized in the structure by hydrogen bond interactions in the oxyanion hole with mainchain amide hydrogens of G143, S144 and C145 (fig. S13b).

Nirmatrelvir resistance associated substitutions

In vitro, Mpro substitutions E166V and L50F+E166V are found to confer a high level of resistance to nirmatrelvir. E166 is a key residue in Mpro connecting the substrate binding site with the dimer interface (*22*). In the substrate binding site, E166 stabilizes substrate binding by mainchain interactions with P3 and side chain interactions to the amide nitrogen in the P1 glutamine (fig. S13a). These interactions are maintained when nirmatrelvir binds (fig. S13b). E166 furthermore interacts with the N-terminal S1 in the other Mpro monomer, and is involved in substrate-induced Mpro dimerization (*22*). As Mpro monomers are not catalytically active, dimerization is essential for Mpro function (*55*).

Additionally, the S1-E166 interaction is essential for maintaining the correct shape of subsite *S1*(*50*). This is supported by several studies of highly similar SARS-CoV Mpro variants. In one

study, it was found that deletion of the N-terminal residues 1-3 in the SARS-CoV Mpro reduced the catalytic efficiency to 76% compared to the original Mpro and led to a slight reduction in dimerization (*56*). In comparison, deletion of N-terminal residues 1-4 led to a dramatic shift of the Mpro monomer-dimer equilibrium resulting in the monomer being the major species and led to a reduction of the catalytic efficiency to 1% (*56*). Another study found that the mutation S1A did not affect the monomer-dimer equilibrium and resulted in 46% catalytic efficiency compared to original Mpro (*57*). A third study found that E166A slightly reduced Mpro dimerization and reduced catalytic efficiency to 31% compared to the original Mpro. In combination with another mutation in the dimer interface, R298A, E166A shifted the Mpro monomer-dimer equilibrium towards monomer as the major species (*22*).

The results in these studies highlight the important role of the S1-E166-substrate interaction for maintaining the enzymatic activity of Mpro. The reduced enzymatic activity upon breakage of the S1-E166-substrate interaction is suggested to result from a disruption of the correct catalytically competent conformation of the substrate binding site including the oxyanion hole (*18*) and a reduction in dimerization (*22*).

Trajectory analysis for simulations

The root-mean-square deviation (RMSD) evolutions of the nirmatrelvir and nsp4/nsp5 substrate peptide extracted from the simulations are shown in fig. S4 and fig. S5, respectively. The RMSD values fluctuate around average values ranging from 1.8-2.4 Å indicating that the Mpro dimer is stable throughout the simulations and that the simulations have converged within 50 ns. We used the last 50 ns of the simulations for further analyses.

Nirmatrelvir-Mpro interactions

To evaluate how the interactions between nirmatrelvir and the SARS-CoV-2 Mpro changed for the Mpro variants, the changes in interaction patterns are illustrated in the heatmap in fig. S6, and Mpro residues for which increased/decreased interactions are observed are shown in fig. S7 and Fig. 7B. In the following, the interaction patterns observed in the MD simulations on the L50F, E166V and L50F+E166V variants compared to the original Mpro are discussed.

The L50F substitution leads to an increase in interactions of R188 and T190 (fig. S6; 15% and 14%, respectively) with the P4 trifluoroacetyl group of nirmatrelvir (fig. S7b and Fig. 7B), and a reduction in interactions of M49 (fig. S6; -13%) with the P2 dimethylcyclopropylproline group of nirmatrelvir (fig. S7b and Fig. 7B).

The E166V substitution leads to a reduction of intermonomer interactions of S1 (fig. S6; -17%) with the P1 γ-lactam ring of nirmatrelvir (fig. S7c and Fig. 7B), and a reduction of interactions of D187 and Q192 (fig. S6; -13% and -15%, respectively) with the P2 dimethylcyclopropylproline group of nirmatrelvir leading to an opening of the binding pocket at subsite *S2* and *S4* as the loop formed by residues 187-192 moves away from nirmatrelvir (fig. S7c and Fig. 7B). We furthermore observe a gain of interactions with L50 (fig. S6; 11%) and the P2 dimethylcyclopropylproline group of nirmatrelvir (fig. S7c and Fig. 7B).

The double substitution L50F+E166V leads to a combination of the changes in interactions observed for the individual substitutions. In comparison with E166V, we observe a more dramatic reduction of intermonomer interactions of S1 (fig. S6; -32%) with the P1 γ-lactam ring of nirmatrelvir (fig. S7d and Fig. 7B). As for L50F, we observe an increase in interactions of R188 and T190 (fig. S6; 27% and 14%, respectively) with the P4 trifluoroacetyl group of nirmatrelvir (fig. S7d and Fig. 7B). These changes in interactions lead to a different binding pose of nirmatrelvir in the binding site, in which the P1 γ-lactam ring moves out of subsite *S1* (formed by F140, L141, H163, V166, H172 in one monomer (monomer A) and S1 in the other monomer (monomer B)), and the nitrile group moves towards T25, L27 and M49 (fig. S7d and Fig. 7B). The reductions of interactions of nirmatrelvir with F140, L141, H163, H172 are -17%, -15%, -14%, and -32%, respectively, and the gains of interactions of nirmatrelvir with T25, L27 and M49 are 11%, 10%, and 12%, respectively.

Interaction energy analysis

The interaction energies extracted from the Mpro-nirmatrelvir and Mpro-substrate peptide MD simulations are presented in table S3, and interaction energy differences are presented in Fig. 7A. The relatively large errors for the substrate peptide simulations reflect the flexibility of the nsp4/nsp5 peptide.

For Mpro with E166V or L50F+E166V, we observe less favourable interaction energies for nirmatrelvir relative to original Mpro resulting mainly from reduction in the coulombic contribution to the interaction energies. This may be explained by the loss of S1-E166-nirmatrelvir interaction (fig. S6). For L50F and L50F+E166V variants, we observe slightly more favourable Lennard-Jones (LJ) energy contributions, which may be explained by a gain in interactions of nirmatrelvir with R188 and T190 (fig. S6).

Inhibition probabilities

For nirmatrelvir to successfully inhibit the SARS-CoV-2 Mpro, nirmatrelvir must bind to the Mpro in a conformation that is compatible with catalysis and thus leads to covalent attachment of nirmatrelvir to Mpro C145. We define a catalytically competent state by two criteria, 1) the sulfur atom in C145 and cyano carbon atom in nirmatrelvir should be in close enough proximity for the nucleophilic attack (*54*), and 2) the backbone amide of G143 in the oxyanion hole and cyano nitrogen atom in nirmatrelvir should be in close enough proximity for stabilizing the nirmatrelvir transition state (*18*). The distances are shown in the Mpro structure in fig. S13 and the numerical values extracted from the simulations are shown as 2D scatter plots in fig. S8. In agreement with other Mpro-substrate peptide/Mpro-nirmatrelvir simulation studies (*54*, *58*), we observe two populations of C145 sulfur and nirmatrelvir cyano carbon distances for the original Mpro. In 24% of the simulation frames, C145 sulfur and nirmatrelvir cyano carbon are in closer proximity than 4.3 Å, and in 76% of the simulation frames further apart than 4.3 Å. In all simulation frames, G143 amide nitrogen and nirmatrelvir cyano nitrogen are in closer proximity than 4.3 Å. Based on these findings, we consider conformations with C145 sulfur-nirmatrelvir cyano carbon distances below 4.3 Å and G143 amide nitrogen and nirmatrelvir cyano nitrogen distances below 4.3 Å as catalytically competent.

For the L50F variant, there is a slight decrease in the probability of nirmatrelvir-Mpro being in a catalytically competent conformation relative to original Mpro (24% to 20%). The probability for E166V to be in a catalytically competent state is reduced to 50% compared to the original Mpro (from 24% to 12%), while a third population with larger G143 amide nitrogen-nirmatrelvir cyano nitrogen distances is present for the L50F+E166V variant thus reducing the probability of a catalytically competent state to 63% compared to the original Mpro (from 24% to 15%).

Impact of mutations on dimerization and integrity of subsite S1

L50F, E166V, and L50F+E166V led to improved or similar Mpro-substrate binding compared to the original Mpro (Fig. 7A). Thus, weakening of substrate binding cannot explain the reduced Mpro activity and replication observed in our experiments (Figs. 4 to 6). Instead, analysis of the Mpro-substrate MD simulations suggests that perturbation of subsite *S1* and destabilization of the Mpro dimer may explain the reduced catalytic activity.

The Mpro exists in two conformations corresponding to an active and an inactive state. The active state of subsite *S1* is stabilized by hydrophobic interactions between F140 and H163 (*21*). We have monitored the distribution of distances between F140 and H163, and their orientation to each other. The latter was defined as the dihedral angle between the phenyl ring of F140 and the imidazole ring

of H163. fig. S10 displays the 2D scatter plots of dihedral angle *vs*. F140-H163 distance for the different variants (original Mpro, E166V, L50F+E166V and L50F). For original Mpro, the most populated state is observed when the distance and dihedral angle between the two side chains is around 4 Å and 0 degrees, respectively, indicating that the side chains are packed face-to-face. This agrees well with other MD simulations on original Mpro in the active conformation (*21*). The distributions for the F140-H163 interaction in E166V and L50F+E166V are broader in comparison to the distributions recorded for original Mpro indicating that the *S1* binding site is more flexible in these variants. This results in the impairment of the hydrophobic stacking between F140 and H163 and consequently inducing structural perturbation leading to the distortion of the active conformation. Even though the substrate can still bind to E166V and L50F+E166V, the Mpro-substrate complex is perturbed leading to an increase in the population of an inactive state of Mpro. For L50F, the distance and orientation between F140 and H163 side chains are not affected by the mutation, resulting in 2D scatter plot resembling the one observed for original Mpro. This observation cannot explain the low activity measured for this variant. We therefore focused on the stability of the Mpro dimer since dimerization is a requirement for Mpro to be catalytically active. We have evaluated the effect of the mutations on dimerization by monitoring the dimer interactions, S1-F140 and R4-E290, which are important for dimer stability (*20*). The results are presented in fig. S9 displaying the 2D scatter plots of the distance R4-E290 *vs*. S1-F140 for the different variants. For original Mpro, the mean S1-F140 distance is 2.8 Å ± 0.1 Å, whereas the mean R4-E290 distance is 3.4 Å ± 1.3 Å. Contrarily, for the variants, relatively large fluctuations in both distances are observed that contribute to the destabilization of the dimer and hence impairment of activity.

Conclusion

In conclusion, we have found that the E166V and L50F+E166V variants gave rise to weakened Mpro-nirmatrelvir binding, whereas the L50F variant gave rise to improved Mpro-nirmatrelvir binding (table S3 and Fig. 7A). On the structural level, the weakened binding (observed in E166V and L50F+E166V variants) is explained by a loss of the enzymatically important S1-E166-nirmatrelvir interaction (fig. S6, S7 and Fig. 7B). For the E166V variant, this loss of interaction was accompanied by a loss of interaction in subsite *S2* (fig. S6) leading to an opening of the binding pocket (fig. S7c and Fig. 7B) and larger distances between the catalytic C145 and the nirmatrelvir cyano group reducing the probability of inhibition by 50 % compared to original Mpro (fig. S8).
For the L50F+E166V variant, the loss of S1-E166-nirmatrelvir interactions and gain in R188 and T190 interactions (fig. S6) led to a conformational change of the nirmatrelvir binding pose in which

the P1 γ-lactam ring moved out of subsite *S1* (fig. S7d and Fig. 7B). Consequently, the distance between C145 in the oxyanion hole and the nirmatrelvir cyano group became too large for catalysis to take place reducing the probability of inhibition by 63% compared to original Mpro (fig. S8). The above analyses of MD simulations provide an atomic-level explanation of the observed resistance against nirmatrelvir observed for the E166V and L50F+E166V variants in cell culture.

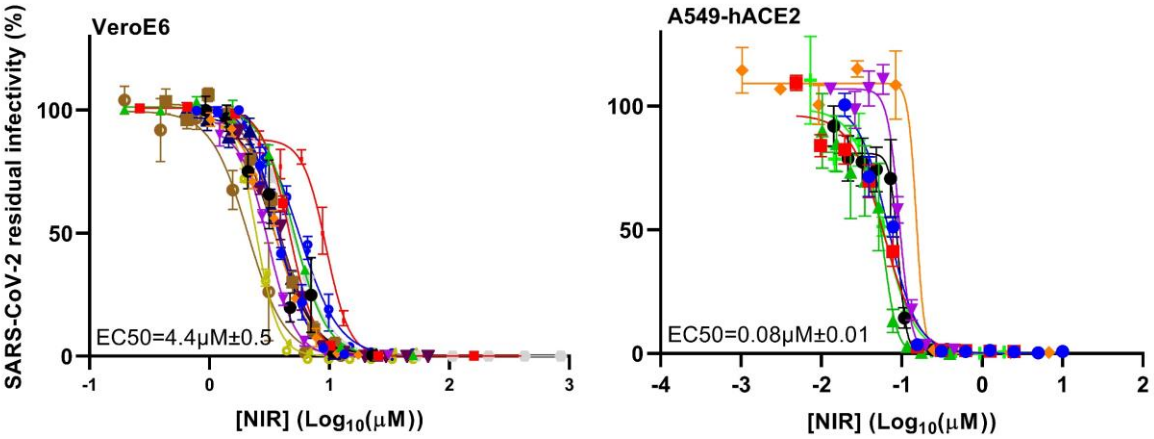

Fig. S1. Determination of EC50 of nirmatrelvir against the original SARS-CoV-2 virus in short-term concentration-response assays. Short-term concentration-response treatments of original SARS-CoV-2 virus with nirmatrelvir (NIR) in VeroE6 or A549-hACE2 cells in 96-well plates. Graphs show all reference treatments carried out during this study and included in the manuscript; different treatments are coded with different colors; 14 and 7 nirmatrelvir treatments were done in VeroE6 and A549-hACE2 cells, respectively. Infected cells were visualized by spike protein immunostaining. Datapoints represent % residual infectivity, calculated by relating the number of infected cells in treated cultures to the mean number of infected cells in at least 8 infected nontreated cultures, and are means of at least 4 replicates ± standard errors of the means (SEM). Curves and 50% effective concentrations (EC50) were determined as described in Online Methods section *Short-term concentration-response treatments*. Given EC50 are the mean of the EC50 of individual curves with SEM and are used as reference EC50 for fold-resistance determinations.

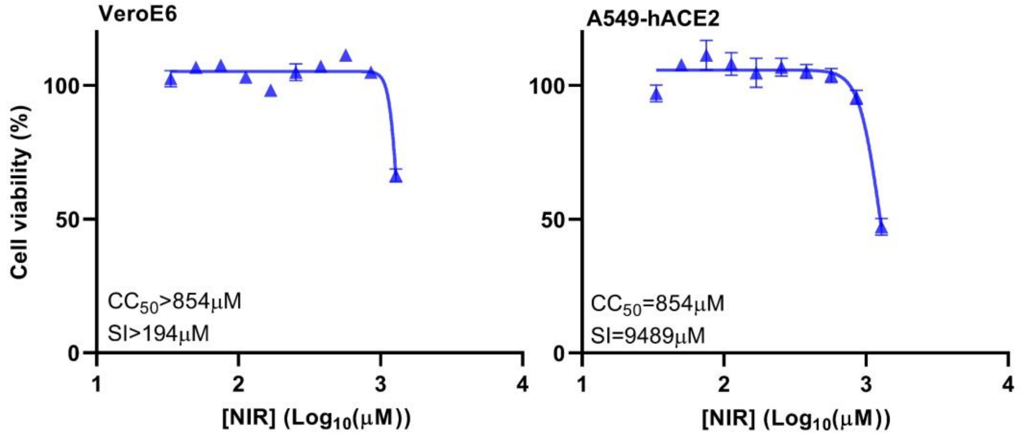

Fig. S2. Nirmatrelvir had a low cytotoxicity and a high selectivity index. Cell viability assays were carried out for nirmatrelvir (NIR) in VeroE6 and A549-hACE2 cells in 96-well plates. Datapoints represent % cell viability, calculated by relating the OD of treated cultures to the mean OD of 10 nontreated cultures, and are means of 3 replicates ± SEM. Curves and 50% cytotoxic concentrations (CC50) were determined using Graphpad Prism 8.0.0 applying the equation Y= Top/(1+10$^{(Log10EC50-X)*HillSlope}$) with a bottom constraint of 0. Selectivity indexes (SI) were determined as CC50/EC50.

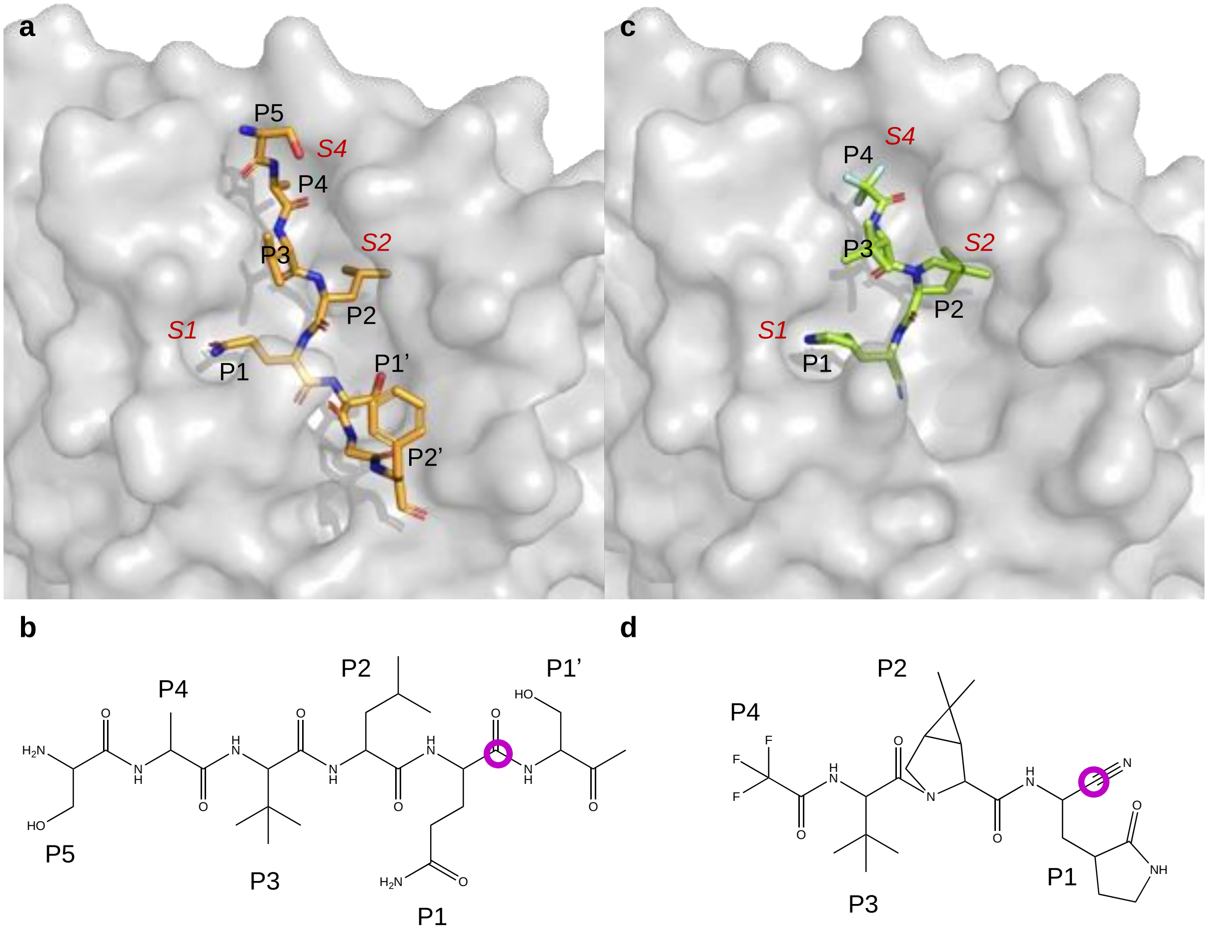

Fig. S3. Structures of Mpro, nsp4-nsp5 substrate and nirmatrelvir. (a) Crystal structure of Mpro (nsp5) with nsp4/nsp5 substrate peptide (orange sticks) bound with residues P5-P1 and P1'-P2' (PDB entry: 7mgs(*33*)). **(b)** Chemical structure of the nsp4/nsp5 junction with residues P5-P1 and P1'. The carbonyl carbon that is the target of the nucleophilic attack by C145 is highlighted with a purple circle. **(c)** Crystal structure of nsp5 with nirmatrelvir (green sticks) covalently bound with labelling of the structural elements mimicking substrate residues P4-P1 (PDB entry: 7vh8(*32*)). The Mpro is shown in a grey surface representation with subsites *S1*, *S2*, and *S4* labelled in red and substrate / nirmatrelvir in stick modus with C: orange/green, N: blue, O:red, F:light-blue in (a) and (c). **(d)** Chemical structure of nirmatrelvir with labelling of the chemical groups mimicking substrate residues P4-P1. The nitrile warhead that is the target of the nucleophilic attack by C145 is highlighted with a purple circle.

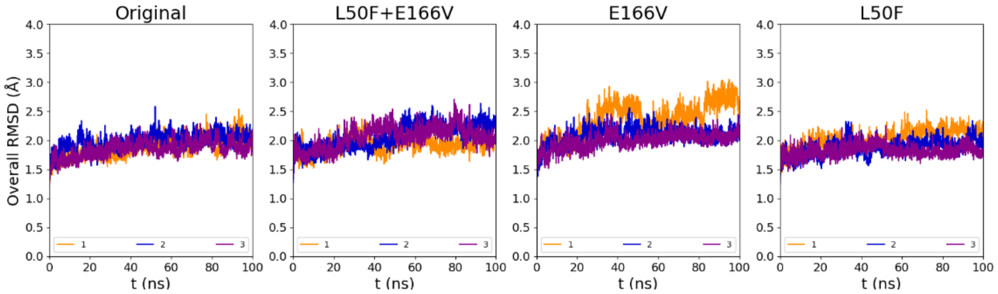

Fig. S4. Time evolution of RMSD for nirmatrelvir simulations. RMSD was calculated for the heavy backbone atoms in Mpro variants relative to the heavy backbone atoms in the corresponding minimized structure. Left to the right: original Mpro; L50F+E166V; E166V; L50F. The three simulation replicates are shown in different colours.

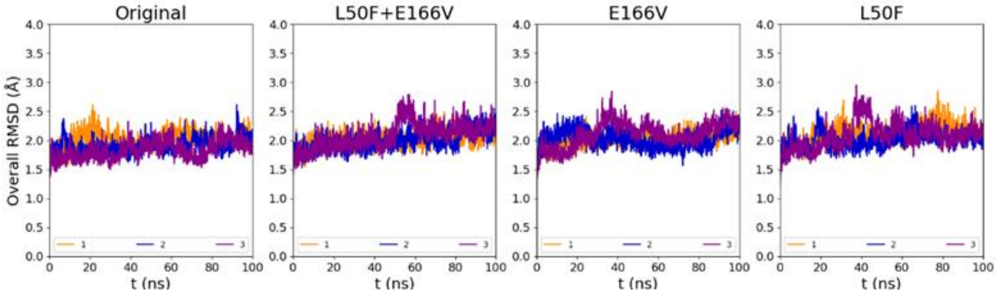

Fig. S5. Time evolution of RMSD for nsp4/nsp5 substrate peptide simulations. RMSD was calculated for the heavy backbone atoms in Mpro variants relative to the heavy backbone atoms in the corresponding minimized structure. Left to the right: original Mpro; L50F+E166V; E166V; L50F. The three simulation replicates are shown in different colours.

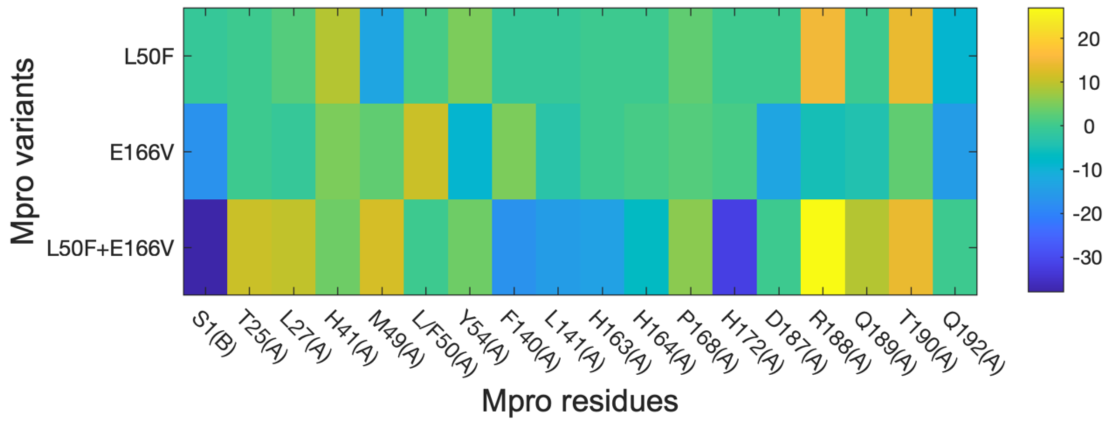

Fig. S6. Influence of Mpro substitutions on interaction of nirmatrelvir with specific Mpro residues. Interaction heatmap showing differences in nirmatrelvir proximities (within 3 Å) between simulations of Mpro variants and original Mpro. The heatmap shows Mpro residues on the x-axis and the studied set of Mpro substitutions on the y-axis. For Mpro residues, (A) signifies location of the residue to Mpro monomer A, while (B) signifies location of the residue to Mpro monomer B. The color bar indicates increased/decreased interactions with respect to the original Mpro. Yellow and dark blue correspond to gain and reduction in interactions, respectively, for the Mpro variants when compared to the original Mpro. Thus, positive and negative percentages indicate that the occurrence of interactions is increased and decreased, respectively, in Mpro variants with substitutions compared to the original Mpro at the given Mpro residues.

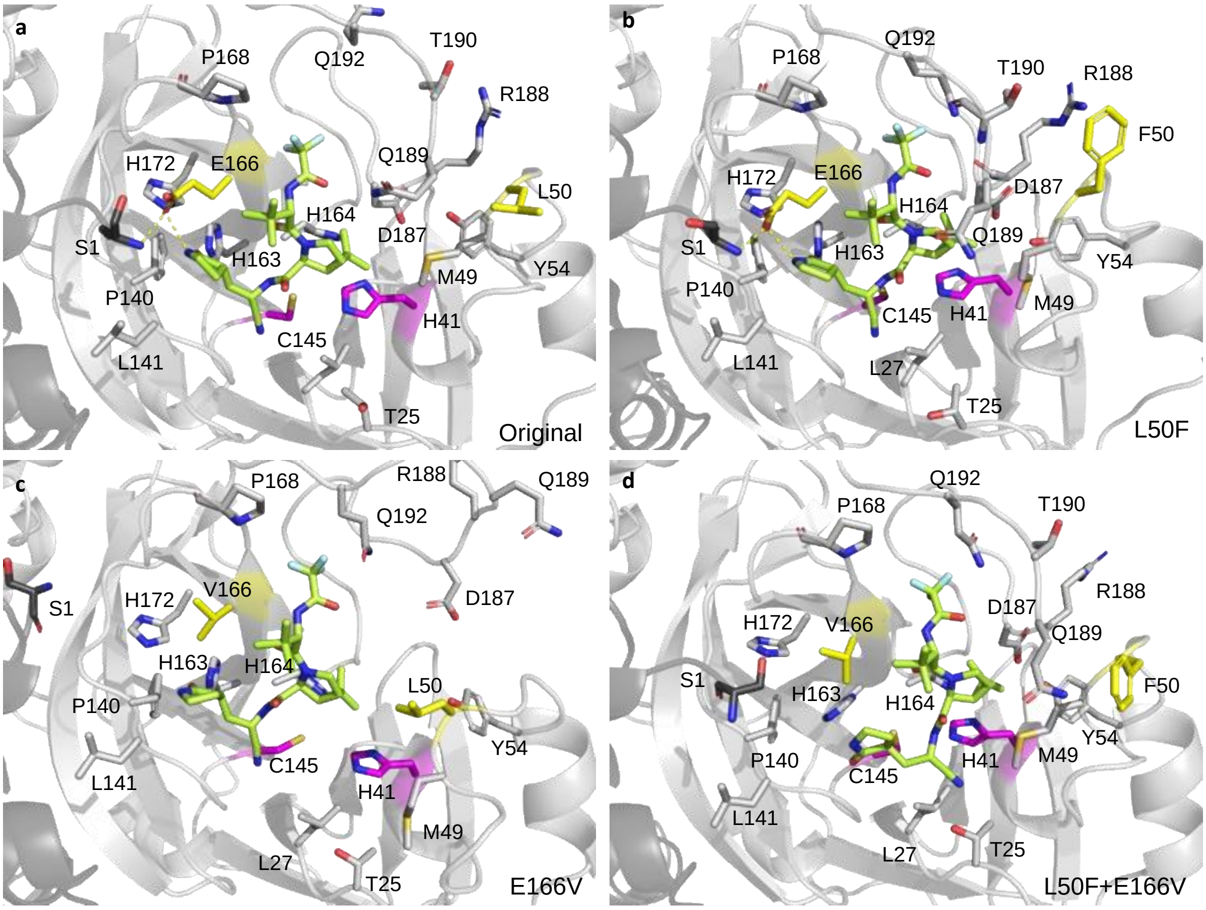

Fig. S7. Mpro-nirmatrelvir conformations for original Mpro and Mpro with resistance associated substitutions. Final Mpro-nirmatrelvir conformations extracted from MD simulations with labelling of Mpro residues for which changes in nirmatrelvir interactions are observed (black) for **(a)** original Mpro, **(b)** L50F, **(c)** E166V, and **(d)** L50F+E166V. The important interactions of E166 in monomer A (light grey) with S1 in monomer B (dark grey) and with nirmatrelvir (green sticks with C:green, N: blue, O:red, F:light-blue) are indicated by yellow dashes. The catalytic dyad (H41 and C145) is shown in magenta and L50F and E166V in yellow.

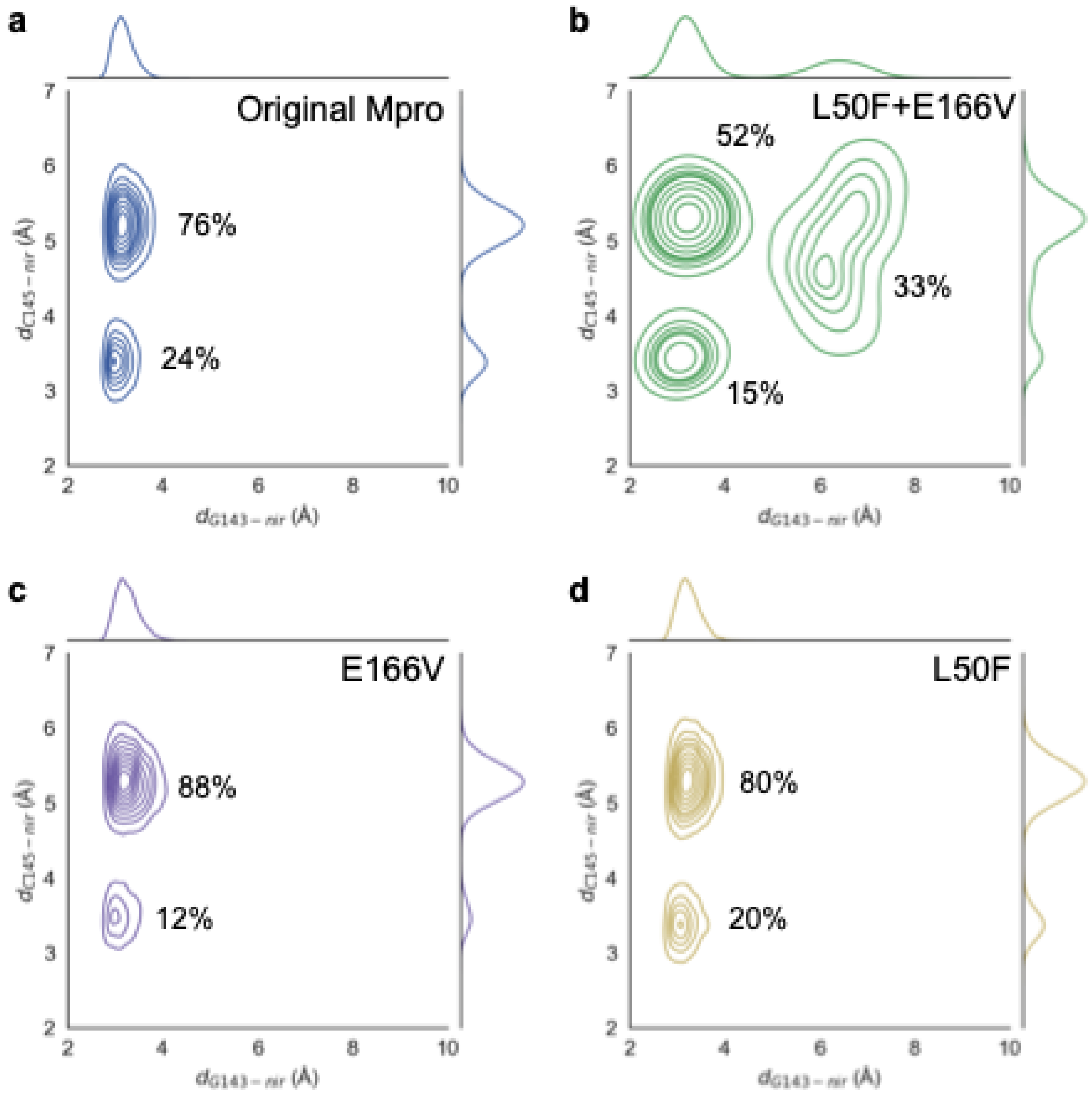

Fig. S8. Shift of Mpro-nirmatrelvir conformational equilibrium for Mpro with resistance associated substitutions. Contour plots with distances between C145 sulfur and nirmatrelvir cyano carbon on the y-axis and between G143 amide nitrogen and nirmatrelvir cyano nitrogen on the x-axis. For each Mpro-nirmatrelvir conformation, the frequency of the conformation is calculated and given in percentage of all simulation frames. a) original Mpro; b) L50F+E166V; c) E166V; d) L50F.

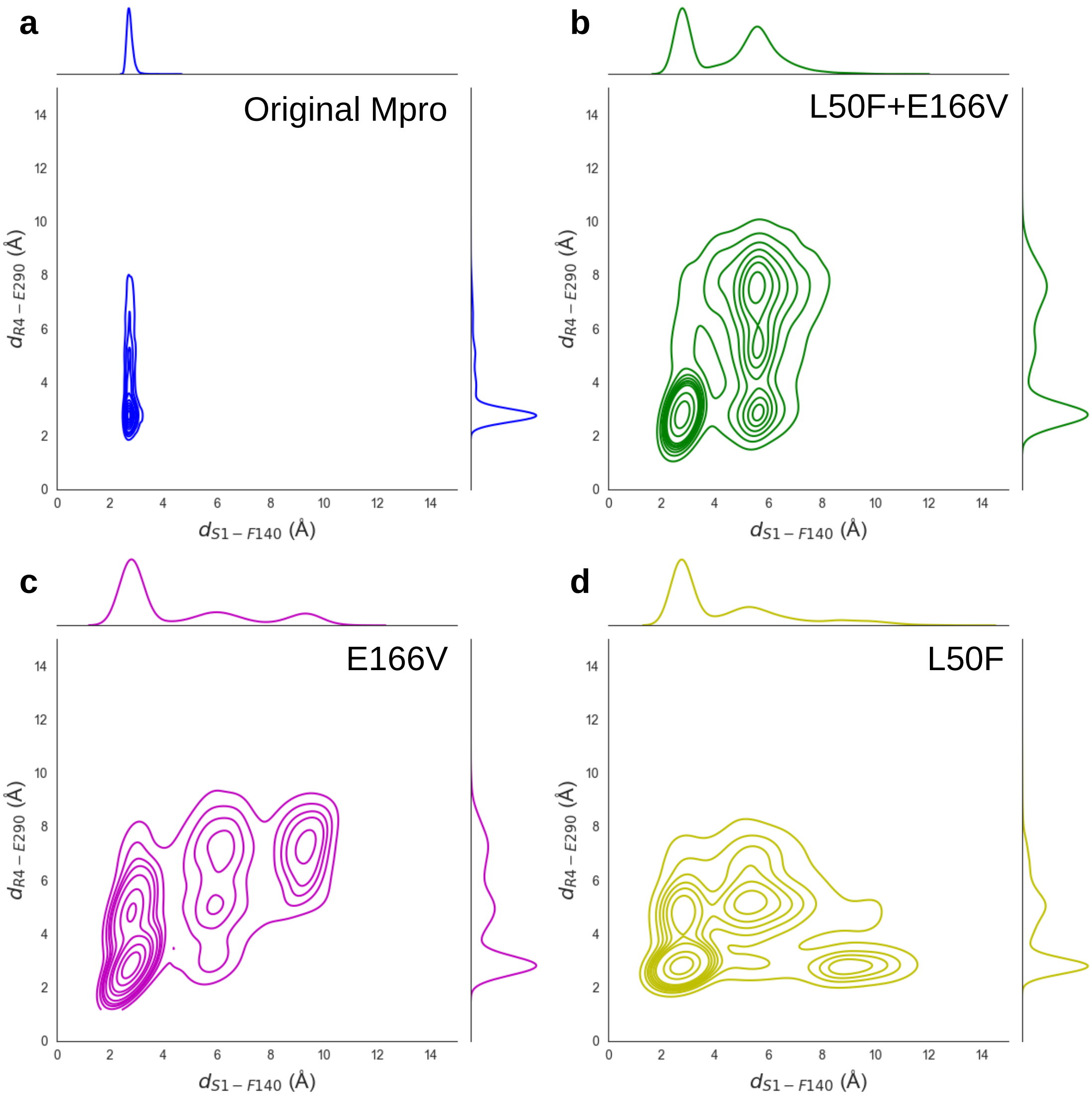

Fig. S9. Disruption of Mpro dimer interactions. Contour plots with closest distances between S1 backbone and F140 backbone on the x-axis and between R4 guanidino group and E290 carboxyl group on the y-axis. a) original Mpro; b) L50F+E166V; c) E166V; d) L50F.

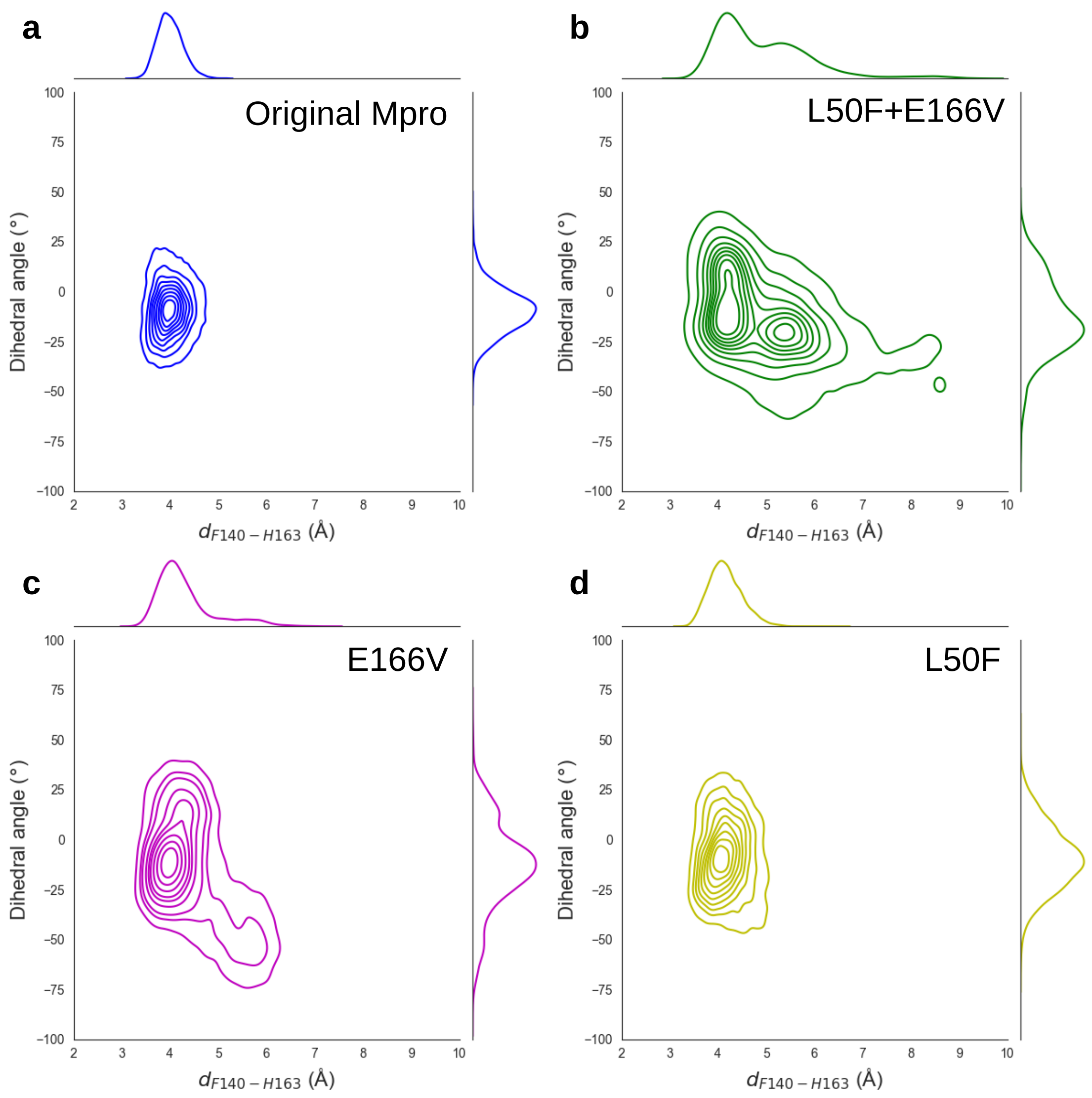

Fig. S10. Disruption of hydrophobic interactions between F140 and H163. Contour plots of dihedral angle *vs*. F140-H163 distance for the different variants. a) original Mpro; b) L50F+E166V; c) E166V; d) L50F.

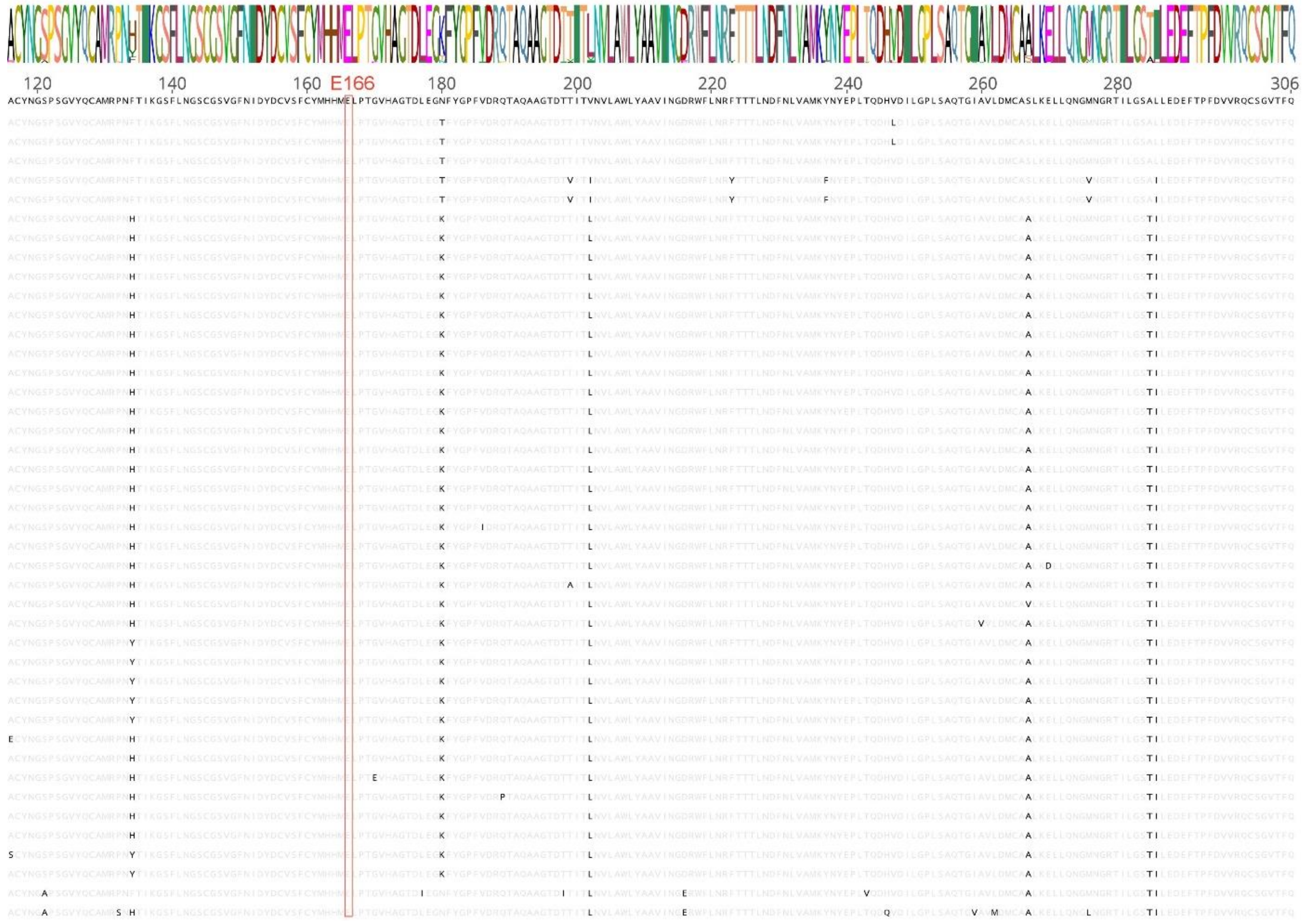
120
140
160
E166
180
200
220
240
260
280
306
ACYNGSPSGVYQCAMRPNFTIKGSFLNGSCGSVGFNIDYDCVSFCYMHHMELPTGVHAGTDLEGNFYGPFVDRQTAQAAGTDTTITVNVLAWLYAAVINGDRWFLNRFTTTLNDFNLVAMKYNYEPLTQDHVDILGPLSAQTGIAVLDMCASLKELLQNGMNGRTILGSALLEDEFTPFDVVRQCSGVTFQ

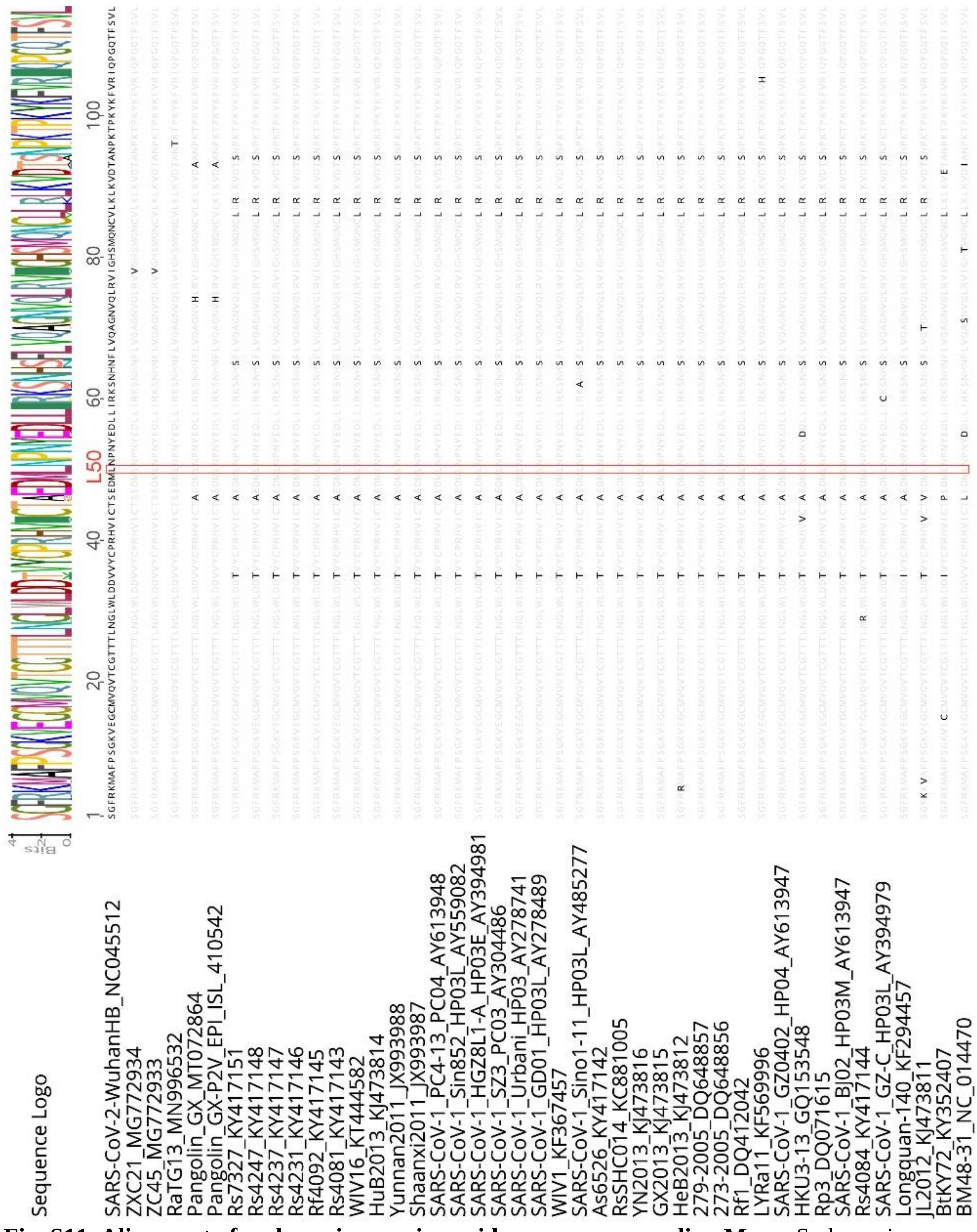

Fig. S11. Alignment of sarbecovirus amino acids sequences encoding Mpro. Sarbecoviruses phylogenetically cluster into four clades based on representative sarbecovirus spike receptor-binding domain amino acid sequences (*49*). 43 representative sabecovirus Mpro sequences from all four clades were chosen for alignment (*49*). The virus names and sequence accession numbers are

listed on the left. Genbank accession numbers are used except for the Pangolin_GX-P2V virus, for which the GISAID accession number is used. The L50 and E166 positions are marked in red and highlighted with red frames.

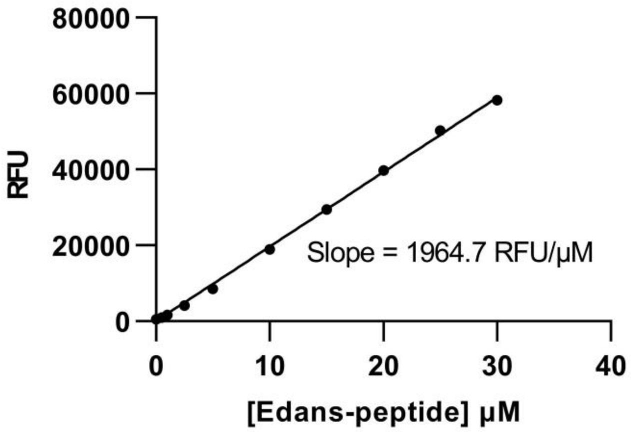

Fig. S12. Edans standard curve for conversion of relative fluorescence units (RFU) to μM. An Edans standard curve was made to convert relative fluorescence units (RFU) to μM in the enzymatic Mpro assays. The standard curve is based on the RFU measurements of known concentrations of cleaved FRET substrate to Edans-peptide.

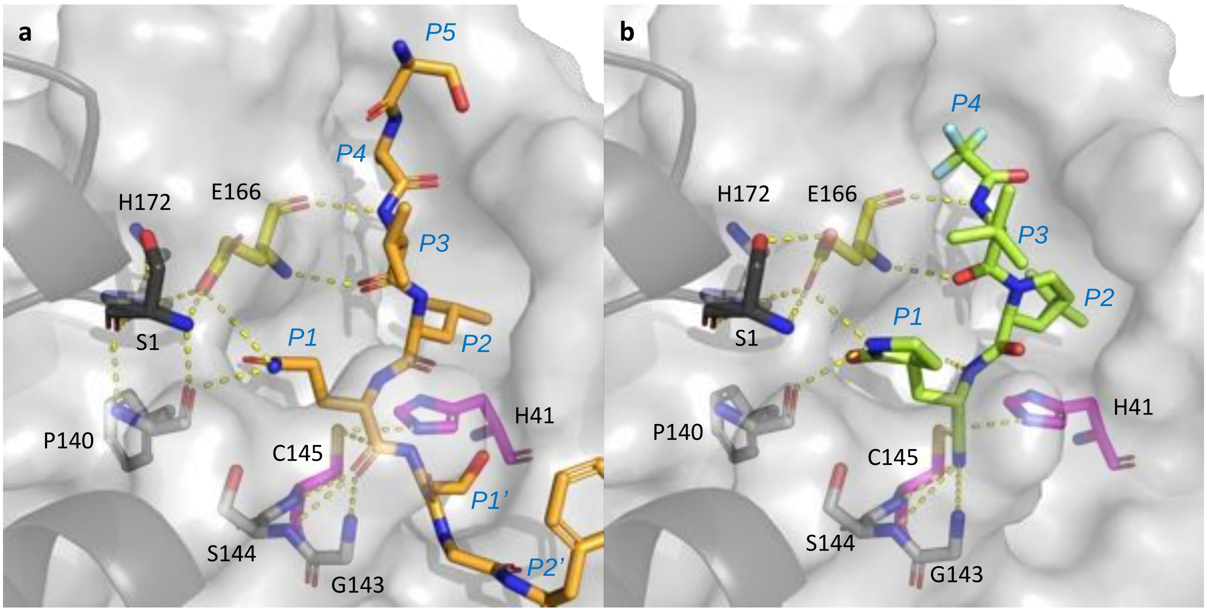

Fig. S13. Structural basis of Mpro-substrate and Mpro-nirmatrelvir interactions. Figure showing important Mpro residues (sticks) and interactions (yellow dashes) for catalysis and binding of **(a)** nsp4/nsp5 substrate peptide (orange sticks) and **(b)** nirmatrelvir (green sticks). E166 (yellow) stabilizes both substrate peptide and nirmatrelvir binding through three hydrogen bonds, and the Mpro dimer through interaction with S1 in the opposite monomer (black). The S1-E166 interaction is a part of a hydrogen bonding network in the *S1* subsite involving, among other residues, F140 and H172. C145 in the catalytic dyad (magenta) is stabilized by a hydrogen bond to H41 and is positioned correctly for a nucleophilic attack on the substrate peptide P1 carbonyl carbon in **(a)** and the nirmatrelvir cyano carbon in **(b)**. Hydrogen bonds from backbone amides of G143, S144, and C145 in the oxyanion hole to the substrate peptide P1 carbonyl oxygen in **(a)** and to the nirmatrelvir cyano nitrogen in **(b)** are shown as yellow dashes.

			Escape 1[a] - %[b]		Escape 2[c] - %[b]					
Nucleotide change[d]	Amino acid change[d]	SARS-CoV-2 protein[e]	6.25xEC50 P1D11[f]	40xEC50 P5D10[f]	6.25xEC50 D17[f]	7.5xEC50 P1D5[f]	10xEC50 P2D3[f]	20xEC50 P3D6[f]	60xEC50 P4D8[f]	120xEC50 P5D9[f]
T834G	F10C	nsp2	-	99	-	-	-	-	-	-
T2827G	N36K	nsp3	-	22	-	-	-	-	-	-
C10116T	T21I	Mpro	-	100	-	-	-	-	-	-
A10197G	D48G	Mpro	-	-	81	-	-	-	-	-
C10202T	L50F	Mpro	-	-	-	11	19	31	98	99
A10551T	E166V	Mpro	-	-	-	99	100	100	99	99
C10965T	T304I	Mpro	99	100	-	-	-	-	-	-
C11379T	A136V	nsp6	-	-	82	99	99	100	100	100
T11522G	F184V	nsp6	-	99	-	-	-	-	-	-
A12497T	N136Y	nsp8	-	-	-	-	-	-	23	11
C13140T	T39I	nsp10	-	-	-	-	-	-	-	-
A15671C	E744A	nsp12	-	-	-	-	41	56	-	-
T17363C	I376T	nsp13	-	-	-	47	79	74	94	96
C19547T	S503L	nsp14	-	-	-	-	16	15	93	95
T24469G	N969K	S	-	-	-	-	-	-	81	96
C26261T	S6L	E	-	-	-	12	-	-	-	-
T26300A	L19H	E	-	-	-	17	48	62	12	10
T26360G	L39W	E	-	-	-	-	-	20	-	-
T27346C	Y49H	ORF6	-	-	-	-	-	-	-	11
A27862G	D36G	ORF7b	-	-	-	-	-	-	36	51
C28697T	P142S	N	-	-	-	-	-	-	36	51

Table S1. Non-synonymous mutations in nirmatrelvir escape viruses in the complete open reading frame (ORF).

[a]Escape 1, source of polyclonal escape virus NIR-EV1: primary escape culture followed by 5 viral passages in VeroE6 cells under treatment with nirmatrelvir. All cultures were treated directly after infection and then every 2-3 days. The primary escape culture was treated with 5xEC50 nirmatrelvir for 7 days. The 1st passage (P1) culture was treated with 6.25xEC50 for 11 days. The 2nd passage (P2) culture was treated with 7.5xEC50 for 5 days. The 3rd passage (P3) culture was treated with 9xEC50 for 3 days. The 4th passage (P4) culture was treated with 20xEC50 for 8 days. The 5th passage culture (P5) was treated with 40xEC50 for 10 days.

[b] Frequency (%) of non-synonymous nucleotide changes in the complete ORF recorded by NGS. Analyzed viruses were harvested from cell culture supernatant. Changes that occurred with at least 10% frequency in at least one of the analyzed virus populations were included in this table. -, frequency of the given change was <10%.

[c]Escape 2, source of polyclonal escape virus NIR-EV2: primary escape culture followed by 5 viral passages in VeroE6 cells under treatment with nirmatrelvir. All cultures were treated directly after infection and then every 2-3 days. The primary escape culture was treated with 6.25xEC50 nirmatrelvir for 30 days. The 1st passage (P1) culture was treated with 7.5xEC50 for 5 days. The 2nd passage (P2) culture was treated with 10xEC50 for 3 days. The 3rd passage (P3) culture was

treated with 20xEC50 for 6 days. The 4th passage (P4) culture was treated with 60xEC50 for 8 days. The 5th passage culture (P5) was treated with 120xEC50 for 9 days.

[d]Nucleotide / amino acid position numbers and original nucleotides / amino acids, given in front of the position numbers, relate to the nucleotide / amino acid sequence of the SARS-CoV-2/human/Denmark/DK-AHH1/2020 strain (GenBank accession number MZ049597). The changed nucleotides / amino acids, acquired during passage under nirmatrelvir treatment, are given after the position numbers.

[e]SARS-CoV-2 protein, to which the identified amino acid changes located relating to the SARS-CoV-2/human/Denmark/DK-AHH1/2020 strain (GenBank accession number MZ049597).

[f]Specification of conditions under which viral genomes subjected to NGS were sampled. Fold EC50 applied and passage (P) and/or day (D) postinfection at sampling time.

Nucleotide change[b]	Amino acid change[b]	SARS-CoV-2 protein[c]	SARS-CoV-2 variant-%[a] T21I P4D2[d]	L50F P4D2[d]	E166V P4D2[d]	T304I P4D2[d]	L50F+ E166V P4D2[d]	T21I+ T304I P4D2[d]
C5183T	P822S	nsp3	-	-	12	-	-	-
C6504A	A1262E	nsp3	-	-	-	17	-	-
A6750G	N1344S	nsp3	27	-	-	-	-	-
A9492C	H313P	nsp4	-	-	87	-	-	-
C10116T	T21I	Mpro	100	-		-	-	100
C10202T	L50F	Mpro	-	100	26	-	100	-
A10551T	E166V	Mpro	-	-	100	-	100	-
C10572T	A173V	Mpro	-	-	-	-	-	-
C10965T	T304I	Mpro	-	-	-	100	-	100
C11379T	A136V	nsp6	-	-	-	-	-	-
C19325T	P429L	nsp14	-	-	-	-	-	-
A22296G	H245R	S	-	28	-	-	-	-
A22206G	D215G	S	-	-	-	-	-	89
C23525T	H655Y	S	-	11	-	-	-	-
G23607A	R682Q	S	-	-	-	14	-	93
G23607T	R682L	S	-	-	-	-	15	-
A23618G	S686G	S	90	-	-	-	-	-
C26261T	S6L	E	-	-	-	-	15	-
C26309A	A22D	E	-	-	-	-	-	-
A27344T	K48I	ORF6	89	-	-	-	-	-

Table S2. Non-synonymous mutations in serially passaged engineered SARS-CoV-2 recombinants in the complete ORF.

[a] Frequency (%) of non-synonymous nucleotide changes in the complete ORF of the specified engineered SARS-CoV-2 recombinants following 4 viral passages in cell culture, as recorded by NGS. Analyzed viruses were harvested from cell culture supernatant. Changes that occurred with at least 10% frequency in at least one of the analyzed virus populations were included in this table. -, frequency of the given change was <10%.

[b]Nucleotide / amino acid position numbers and original nucleotides / amino acids, given in front of the position numbers, relate to the nucleotide / amino acid sequence of the SARS-CoV-2/human/Denmark/DK-AHH1/2020 strain (GenBank accession number MZ049597). The changed nucleotides / amino acids, acquired following serial passage, are given after the position numbers.

[c]SARS-CoV-2 protein, to which the identified amino acid changes located relating to the SARS-CoV-2/human/Denmark/DK-AHH1/2020 strain (GenBank accession number MZ049597).

[d]Specification of the SARS-CoV-2 variant and the time when viral genomes subjected to NGS were sampled, being passage 4 (P4) day 2 (D2) postinfection for all variants.

	Nirmatrelvir						Substrate peptide					
	Coul.		LJ		Total		Coul.		LJ		Total	
**Original**	-160	±1	-170	±4	-330	±3	-227	±14	-239	±6	-466	±18
**L50F**	-163	±2	-180	±8	-343	±6	-261	±17	-234	±12	-495	±24
**E166V**	-147	±2	-170	±4	-317	±6	-241	±12	-250	±5	-491	±15
**L50F+E166V**	-138	±4	-175	±5	-313	±5	-232	±8	-252	±7	-484	±13

Table S3. Overview of interaction energies extracted from the MD simulations for original, L50F, E166V, and L50F+E166V Mpro given in kJ/mol with nirmatrelvir or nsp4/nsp5 substrate peptide bound, respectively. Total interaction energies are given as well as coulombic (Coul.) and Lennard-Jones (LJ) contributions. The energies given in the table are averages of three replicates, and the uncertainties are standard errors of mean.

	Amino acid residue[b]	SARS-CoV-2 Mpro residue[a]			
		L50	E166	T21	T304
Number of viruses[c] (Total: 10,302,924 viruses)	A	3	5	122	21
	R	20	7	0	1
	N	0	4	80	54
	D	27	86	13	2
	C	1	4	1	0
	Q	0	4716	1	0
	E	0	0	0	0
	G	2	17	0	0
	H	13	130	1	1
	I	38	1	15255	836
	L	0	3	3	1
	K	11	7	0	1
	M	1	5	1	0
	F	4370	4	0	0
	P	11	1	3	4
	S	97	11	18	3
	T	2	11	0	0
	W	0	0	0	0
	Y	0	2	0	4
	V	39	5	6	1
	del	26	70	13	1
	Number of substitutions[d]	4650	5082	15517	929

Table S4. Naturally occurring substitutions in SARS-CoV-2 Mpro.

[a]SARS-CoV-2 Mpro residues of interest due to identification of resistance associated substitutions in this study.

[b]Amino acid identity using one letter codes; del, deletion.

[c]For this analysis, a total of 10,302,924 SARS-CoV-2 sequences were retrived from the GISAID database on April 18th, 2022.

[d]Total number of viruses with any substitution at L50, E166, T21 or T304 in Mpro.

Nucleotide change[e]	Amino acid change[e]	SARS-CoV-2 protein[f]	L50F Escape 1[a] - %[b] 5xEC50 D9[g]	L50F Escape 1[a] - %[b] 10xEC50 P1D5[g]	L50F Escape 2[c] - %[b] 5.25xEC50 D9[g]	L50F Escape 2[c] - %[b] 10xEC50 P1D5[g]	L50F Escape 3[d] - %[b] 5.5xEC50 D9[g]	L50F Escape 3[d] - %[b] 10xEC50 P1D5[g]
G7387A	M1556I	nsp3	-	-	-	-	-	16
T8166C	I1816T	nsp3	-	-	98	100	-	-
C10202T	L50F	Mpro	100	100	100	100	99	100
T10484G	S144A	Mpro	-	-	-	97	-	-
A10551C	E166A	Mpro	-	-	-	-	-	99
A10551T	E166V	Mpro	-	99	-	-	-	-
C11511T	T180I	nsp6	-	-	-	-	-	-
T11522G	F184V	nsp6	-	-	-	-	-	-
A16514G	Y93C	nsp13	-	-	-	-	-	-
C17678T	T481M	nsp13	-	-	-	-	-	-
C18788T	T250I	nsp14	-	-	99	100	-	-
C23606T	R682W	S	-	-	81	99	-	-
C26333T	T30I	E	-	-	-	-	-	-
A26760G	I80V	M	-	-	-	-	-	-
A28065G	I58V	ORF8	-	-	-	-	-	-
A29237G	M322V	N	-	-	-	-	-	32

Table S5. Non-synonymous mutations in nirmatrelvir escape viruses with pre-existing L50F in the complete ORF.

[a]L50F Escape 1: primary escape culture followed by 1 viral passage in VeroE6 cells under treatment with nirmatrelvir. All cultures were treated directly after infection and then every 2-3 days. The primary escape culture was treated with 5xEC50 nirmatrelvir for 11 days. The 1st passage (P1) culture was treated with 10xEC50 for 5 days.

[b] Frequency (%) of non-synonymous nucleotide changes in the complete ORF recorded by NGS. Analyzed viruses were harvested from cell culture supernatant. Changes that occurred with at least 10% frequency in at least one of the analyzed virus populations were included in this table. -, frequency of the given change was <10%.

[c]L50F Escape 2: primary escape culture followed by 1 viral passage in VeroE6 cells under treatment with nirmatrelvir. All cultures were treated directly after infection and then every 2-3 days. The primary escape culture was treated with 5.25xEC50 nirmatrelvir for 11 days. The 1st passage (P1) culture was treated with 10xEC50 for 5 days.

[d]L50F Escape 3: primary escape culture followed by 1 viral passage in VeroE6 cells under treatment with nirmatrelvir. All cultures were treated directly after infection and then every 2-3 days. The primary escape culture was treated with 5.5xEC50 nirmatrelvir for 11 days. The 1st passage (P1) culture was treated with 10xEC50 for 5 days.

[e]Nucleotide / amino acid position numbers and original nucleotides / amino acids, given in front of the position numbers, relate to the nucleotide / amino acid sequence of the SARS-CoV-2/human/Denmark/DK-AHH1/2020 strain (GenBank accession number MZ049597). The changed

nucleotides / amino acids, acquired during passage under nirmatrelvir treatment, are given after the position numbers.

[f]SARS-CoV-2 protein, to which the identified amino acid changes located relating to the SARS-CoV-2/human/Denmark/DK-AHH1/2020 strain (GenBank accession number MZ049597).

[g]Specification of conditions under which viral genomes subjected to NGS were sampled. Fold EC50 applied and passage (P) and/or day (D) postinfection at sampling time.

REFERENCES AND NOTES

1. COVID-19: EMA recommends conditional marketing authorisation for Paxlovid | European Medicines Agency; www.ema.europa.eu/en/news/covid-19-ema-recommends-conditional-marketing-authorisation-paxlovid.

2. Fact sheet for healthcare providers: Emergency use authorization for Paxlovid™ highlights of emergency use authorization; www.fda.gov/media/155050/download.

3. J. Hammond, H. Leister-Tebbe, A. Gardner, P. Abreu, W. Bao, W. Wisemandle, M. Baniecki, V. M. Hendrick, B. Damle, A. Simón-Campos, R. Pypstra, J. M. Rusnak, Oral nirmatrelvir for high-risk, nonhospitalized adults with Covid-19. *N. Engl. J. Med.* **386**, 1397–1408 (2022).

4. A. Jayk Bernal, M. M. Gomes da Silva, D. B. Musungaie, E. Kovalchuk, A. Gonzalez, V. Delos Reyes, A. Martín-Quirós, Y. Caraco, A. Williams-Diaz, M. L. Brown, J. Du, A. Pedley, C. Assaid, J. Strizki, J. A. Grobler, H. H. Shamsuddin, R. Tipping, H. Wan, A. Paschke, J. R. Butterton, M. G. Johnson, C. De Anda; MOVe-OUT Study Group, Molnupiravir for oral treatment of Covid-19 in nonhospitalized patients. *N. Engl. J. Med.* **386**, 509–520 (2022).

5. Fact sheet for patients and caregivers emergency use authorization (EUA) of Lagevrio™ (molnupiravir) capsules for coronavirus disease 2019 (COVID-19); www.fda.gov/media/155055/download.

6. R. L. Gottlieb, C. E. Vaca, R. Paredes, J. Mera, B. J. Webb, G. Perez, G. Oguchi, P. Ryan, B. U. Nielsen, M. Brown, A. Hidalgo, Y. Sachdeva, S. Mittal, O. Osiyemi, J. Skarbinski, K. Juneja, R. H. Hyland, A. Osinusi, S. Chen, G. Camus, M. Abdelghany, S. Davies, N. Behenna-Renton, F. Duff, F. M. Marty, M. J. Katz, A. A. Ginde, S. M. Brown, J. T. Schiffer, J. A. Hill, Early remdesivir to prevent progression to Severe Covid-19 in outpatients. *N. Engl. J. Med.* **386**, 305–315 (2022).

7. Veklury | European Medicines Agency; www.ema.europa.eu/en/medicines/human/EPAR/veklury.

8. Fact sheet for health care providers emergency use authorization (EUA) of Veklury® (remdesivir); www.samc.com/assets/documents/covid19/nursing/remdesivir_eua-hcp-fact-sheet-8-2020.pdf.

9. J. H. Beigel, K. M. Tomashek, L. E. Dodd, A. K. Mehta, B. S. Zingman, A. C. Kalil, E. Hohmann, H. Y. Chu, A. Luetkemeyer, S. Kline, D. Lopez de Castilla, R. W. Finberg, K. Dierberg, V. Tapson, L. Hsieh, T. F. Patterson, R. Paredes, D. A. Sweeney, W. R. Short, G. Touloumi, D. C. Lye, N. Ohmagari, M. Oh, G. M. Ruiz-Palacios, T. Benfield, G. Fätkenheuer, M. G. Kortepeter, R. L. Atmar, C. B. Creech, J. Lundgren, A. G. Babiker, S. Pett, J. D. Neaton, T. H. Burgess, T. Bonnett, M. Green, M. Makowski, A. Osinusi, S. Nayak, H. C. Lane, Remdesivir for the treatment of Covid-19—Final report. *N. Engl. J. Med.* **383**, 1813–1826 (2020).

10. S. Iketani, L. L. Liu, Y. Guo, L. L. Liu, J. F.-W. Chan, Y. Y. Huang, M. Wang, Y. Luo, J. Yu, H. Chu, K. K.-H. Chik, T. T.-T. Yuen, M. T. Yin, M. E. Sobieszczyk, Y. Y. Huang, K.-Y. Yuen, H. H. Wang, Z. Sheng, D. D. Ho, Antibody evasion properties of SARS-CoV-2 Omicron sublineages. *Nature* **604**, 553–556 (2022).

11. Fact sheet for healthcare providers: Emergency use authorization for bebtelovimab highlights of emergency use authorization (EUA). These highlights of the EUA do not include all the information needed to use BEBTELOVIMAB under the EUA; www.fda.gov/media/156152/download.

12. M. J. Peluso, K. Anglin, M. S. Durstenfeld, J. N. Martin, J. D. Kelly, P. Y. Hsue, T. J. Henrich, S. G. Deeks, Effect of oral nirmatrelvir on long COVID Symptoms: 4 cases and rationale for systematic studies. *Pathog. Immun.* **7**, 95–103 (2022).

13. S. Mason, J. P. Devincenzo, S. Toovey, J. Z. Wu, R. J. Whitley, Comparison of antiviral resistance across acute and chronic viral infections. *Antiviral Res.* **158**, 103–112 (2018).

14. S. Ramirez, C. Fernandez-Antunez, A. Galli, A. Underwood, L. V. Pham, L. A. Ryberg, S. Feng, M. S. Pedersen, L. S. Mikkelsen, S. Belouzard, J. Dubuisson, C. Sølund, N. Weis, J. M. Gottwein, U. Fahnøe, J. Bukh, Overcoming culture restriction for SARS-CoV-2 in human

cells facilitates the screening of compounds inhibiting viral replication. *Antimicrob. Agents Chemother.* **65**, e0009721 (2021).

15. K. A. Gammeltoft, Y. Zhou, C. R. D. Hernandez, A. Galli, A. Offersgaard, R. Costa, L. V. Pham, U. Fahnøe, S. Feng, T. K. H. Scheel, S. Ramirez, J. Bukh, J. M. Gottwein, Hepatitis C virus protease inhibitors show differential efficacy and interactions with remdesivir for treatment of SARS-CoV-2 in vitro. *Antimicrob. Agents Chemother.* **65**, e0268020 (2021).

16. D. R. Owen, C. M. N. Allerton, A. S. Anderson, L. Aschenbrenner, M. Avery, S. Berritt, B. Boras, R. D. Cardin, A. Carlo, K. J. Coffman, A. Dantonio, L. Di, H. Eng, R. A. Ferre, K. S. Gajiwala, S. A. Gibson, S. E. Greasley, B. L. Hurst, E. P. Kadar, A. S. Kalgutkar, J. C. Lee, J. Lee, W. Liu, S. W. Mason, S. Noell, J. J. Novak, R. S. Obach, K. Ogilvie, N. C. Patel, M. Pettersson, D. K. Rai, M. R. Reese, M. F. Sammons, J. G. Sathish, R. S. P. Singh, C. M. Steppan, A. E. Stewart, J. B. Tuttle, L. Updyke, P. R. Verhoest, L. Wei, Q. Yang, Y. Zhu, An oral SARS-CoV-2 Mpro inhibitor clinical candidate for the treatment of COVID-19. *Science* **374**, 1586–1593 (2021).

17. U. Fahnøe, L. V. Pham, C. Fernandez-Antunez, R. Costa, L. R. Rivera-Rangel, A. Galli, S. Feng, L. S. Mikkelsen, J. M. Gottwein, T. K. H. Scheel, S. Ramirez, J. Bukh, Versatile SARS-CoV-2 reverse-genetics systems for the study of antiviral resistance and replication. *Viruses* **14**, 172 (2022).

18. J. Tan, K. H. G. Verschueren, K. Anand, J. Shen, M. Yang, Y. Xu, Z. Rao, J. Bigalke, B. Heisen, J. R. Mesters, K. Chen, X. Shen, H. Jiang, R. Hilgenfeld, pH-dependent conformational flexibility of the SARS-CoV main proteinase (M(pro)) dimer: Molecular dynamics simulations and multiple x-ray structure analyses. *J. Mol. Biol.* **354**, 25–40 (2005).

19. J. M. Flynn, N. Samant, G. Schneider-Nachum, D. T. Barkan, N. K. Yilmaz, C. A. Schiffer, S. A. Moquin, D. Dovala, D. N. A. Bolon, Comprehensive fitness landscape of SARS-CoV-2 Mpro reveals insights into viral resistance mechanisms. *eLife* **11**, e77433 (2022).

20. B. Goyal, D. Goyal, Targeting the dimerization of the main protease of coronaviruses: A potential broad-spectrum therapeutic strategy. *ACS Comb. Sci.* **22**, 297–305 (2020).

21. C. Li, X. Teng, Y. Qi, B. Tang, H. Shi, X. Ma, L. Lai, Conformational flexibility of a short loop near the active site of the SARS-3CLpro is essential to maintain catalytic activity. *Sci. Rep.* **6**, 20918 (2016).

22. S. C. Cheng, G. G. Chang, C. Y. Chou, Mutation of Glu-166 blocks the substrate-induced dimerization of SARS coronavirus main protease. *Biophys. J.* **98**, 1327–1336 (2010).

23. E. Heilmann, F. Costacurta, S. A. Moghadasi, C. Ye, M. Pavan, D. Bassani, A. Volland, C. Ascher, A. K. H. Weiss, D. Bante, R. S. Harris, S. Moro, B. Rupp, L. Martinez-Sobrido, D. von Laer, SARS-CoV-2 3CL pro mutations selected in a VSV-based system confer resistance to nirmatrelvir, ensitrelvir, and GC376. *Sci. Transl. Med.*, eabq7360 (2022).

24. M. E. Charness, K. Gupta, G. Stack, J. Strymish, E. Adams, D. C. Lindy, H. Mohri, D. D. Ho, Rebound of SARS-CoV-2 infection after nirmatrelvir-ritonavir treatment. *N. Engl. J. Med.* **387**, 1045–1047 (2022).

25. A. S. Anderson, P. Caubel, J. M. Rusnak, Nirmatrelvir-ritonavir and viral load rebound in Covid-19. *N. Engl. J. Med.* **387**, 1047–1049 (2022).

26. Y. Zhou, K. Gilmore, S. Ramirez, E. Settels, K. A. Gammeltoft, L. V. Pham, U. Fahnøe, S. Feng, A. Offersgaard, J. Trimpert, J. Bukh, K. Osterrieder, J. M. Gottwein, P. H. Seeberger, In vitro efficacy of artemisinin-based treatments against SARS-CoV-2. *Sci. Rep.* **11**, 14571 (2021).

27. Y. Zhou, K. A. Gammeltoft, A. Galli, A. Offersgaard, U. Fahnøe, S. Ramirez, J. Bukh, J. M. Gottwein, Efficacy of ion-channel inhibitors amantadine, memantine and rimantadine for the treatment of SARS-CoV-2 in vitro. *Viruses* **13**, 2082 (2021).

28. C. Sølund, A. P. Underwood, C. Fernandez-Antunez, S. Bollerup, L. S. Mikkelsen, S. L. Villadsen, U. Fahnøe, A. A. Winckelmann, S. Feng, C. A. Nørløv Vinten, M. I. Dalegaard, G. Vizgirda, A. L. Sørensen, S. Ramirez, J. Bukh, N. Weis, Analysis of neutralization titers against SARS-CoV-2 in health-care workers vaccinated with prime-boost mRNA-mRNA or vector-mRNA COVID-19 vaccines. *Vaccine* **10**, 75 (2022).

29. V. M. Corman, O. Landt, M. Kaiser, R. Molenkamp, A. Meijer, D. K. W. Chu, T. Bleicker, S. Brünink, J. Schneider, M. L. Schmidt, D. G. J. C. Mulders, B. L. Haagmans, B. Van Der Veer, S. Van Den Brink, L. Wijsman, G. Goderski, J. L. Romette, J. Ellis, M. Zambon, M. Peiris, H. Goossens, C. Reusken, M. P. G. Koopmans, C. Drosten, Detection of 2019 novel coronavirus (2019-nCoV) by real-time RT-PCR. *Eurosurveillance* **25**, 2000045 (2020).

30. A. Offersgaard, C. R. D. Hernandez, A. F. Pihl, R. Costa, N. P. Venkatesan, X. Lin, L. Van Pham, S. Feng, U. Fahnøe, T. K. H. Scheel, S. Ramirez, U. Reichl, J. Bukh, Y. Genzel, J. M. Gottwein, SARS-CoV-2 production in a scalable high cell density bioreactor. *Vaccine* **9**, 706 (2021).

31. C. Ma, M. D. Sacco, B. Hurst, J. A. Townsend, Y. Hu, T. Szeto, X. Zhang, B. Tarbet, M. T. Marty, Y. Chen, J. Wang, Boceprevir, GC-376, and calpain inhibitors II, XII inhibit SARS-CoV-2 viral replication by targeting the viral main protease. *Cell Res.* **30**, 678–692 (2020).

32. Y. Zhao, C. Fang, Q. Zhang, R. Zhang, X. Zhao, Y. Duan, H. Wang, Y. Zhu, L. Feng, J. Zhao, M. Shao, X. Yang, L. Zhang, C. Peng, K. Yang, D. Ma, Z. Rao, H. Yang, Crystal structure of SARS-CoV-2 main protease in complex with protease inhibitor PF-07321332. *Protein Cell* **13**, 689–693 (2022).

33. E. A. MacDonald, G. Frey, M. N. Namchuk, S. C. Harrison, S. M. Hinshaw, I. W. Windsor, Recognition of divergent viral substrates by the SARS-CoV-2 main protease. *ACS Infect. Dis.* **7**, 2591–2595 (2021).

34. R. Anandakrishnan, B. Aguilar, A. V. Onufriev, H++ 3.0: Automating pK prediction and the preparation of biomolecular structures for atomistic molecular modeling and simulations. *Nucleic Acids Res.* **40**, W537–W541 (2012).

35. J. D. Cortese, A. L. Voglino, C. R. Hackenbrock, The ionic strength of the intermembrane space of intact mitochondria is not affected by the pH or volume of the intermembrane space. *Biochim. Biophys. Acta* **1100**, 189–197 (1992).

36. R. B. Best, X. Zhu, J. Shim, P. E. M. Lopes, J. Mittal, M. Feig, A. D. MacKerell, Optimization of the additive CHARMM all-atom protein force field targeting improved sampling of the backbone φ, ψ and side-chain χ(1) and χ(2) dihedral angles. *J. Chem. Theory Comput.* **8**, 3257–3273 (2012).

37. J. Huang, S. Rauscher, G. Nawrocki, T. Ran, M. Feig, B. L. De Groot, H. Grubmüller, A. D. MacKerell, CHARMM36m: An improved force field for folded and intrinsically disordered proteins. *Nat. Methods* **14**, 71–73 (2017).

38. K. Vanommeslaeghe, E. Hatcher, C. Acharya, S. Kundu, S. Zhong, J. Shim, E. Darian, O. Guvench, P. Lopes, I. Vorobyov, A. D. Mackerell, CHARMM general force field: A force field for drug-like molecules compatible with the CHARMM all-atom additive biological force fields. *J. Comput. Chem.* **31**, 671–690 (2010).

39. W. Yu, X. He, K. Vanommeslaeghe, A. D. MacKerell, Extension of the CHARMM General Force Field to sulfonyl-containing compounds and its utility in biomolecular simulations. *J. Comput. Chem.* **33**, 2451–2468 (2012).

40. D. Van Der Spoel, E. Lindahl, B. Hess, G. Groenhof, A. E. Mark, H. J. C. Berendsen, GROMACS: Fast, flexible, and free. *J. Comput. Chem.* **26**, 1701–1718 (2005).

41. M. J. Abraham, T. Murtola, R. Schulz, S. Páll, J. C. Smith, B. Hess, E. Lindah, GROMACS: High performance molecular simulations through multi-level parallelism from laptops to supercomputers. *SoftwareX* **1-2**, 19–25 (2015).

42. H. J. C. Berendsen, J. P. M. Postma, W. F. Van Gunsteren, A. Dinola, J. R. Haak, Molecular dynamics with coupling to an external bath. *J. Chem. Phys.* **81**, 3684 (1998).

43. M. Parrinello, A. Rahman, Polymorphic transitions in single crystals: A new molecular dynamics method. *J. Appl. Phys.* **52**, 7182 (1998).

44. S. Nosé, M. L. Klein, Constant pressure molecular dynamics for molecular systems. *Mol. Phys.* **50**, 1055–1076 (1983).

45. U. Essmann, L. Perera, M. L. Berkowitz, T. Darden, H. Lee, L. G. Pedersen, A smooth particle mesh Ewald method. *J. Chem. Phys.* **103**, 8577 (1998).

46. T. Darden, D. York, L. Pedersen, Particle mesh Ewald: An N·log(N) method for Ewald sums in large systems. *J. Chem. Phys.* **98**, 10089 (1998).

47. B. Hess, H. Bekker, H. J. C. Berendsen, J. G. E. M. Fraaije, LINCS: A linear constraint solver for molecular simulations. *J. Comput. Chem.* **18**, 14631472 (1997).

48. W. Humphrey, A. Dalke, K. Schulten, VMD: Visual molecular dynamics. *J. Mol. Graph.* **14**, 33–38 (1996).

49. T. N. Starr, S. K. Zepeda, A. C. Walls, A. J. Greaney, S. Alkhovsky, D. Veesler, J. D. Bloom, ACE2 binding is an ancestral and evolvable trait of sarbecoviruses. *Nature* **603**, 913–918 (2022).

50. L. Zhang, D. Lin, X. Sun, U. Curth, C. Drosten, L. Sauerhering, S. Becker, K. Rox, R. Hilgenfeld, Crystal structure of SARS-CoV-2 main protease provides a basis for design of improved α-ketoamide inhibitors. *Science* **368**, 409–412 (2020).

51. J. A. Mótyán, M. Mahdi, G. Hoffka, J. Tőzsér, Potential resistance of SARS-CoV-2 main protease (Mpro) against protease inhibitors: Lessons learned from HIV-1 protease. *Int. J. Mol. Sci.* **23**, 3507 (2022).

52. J. Lee, L. J. Worrall, M. Vuckovic, F. I. Rosell, F. Gentile, A. T. Ton, N. A. Caveney, F. Ban, A. Cherkasov, M. Paetzel, N. C. J. Strynadka, Crystallographic structure of wild-type SARS-CoV-2 main protease acyl-enzyme intermediate with physiological C-terminal autoprocessing site. *Nat. Commun.* **11**, 1–9 (2020).

53. K. Świderek, V. Moliner, Revealing the molecular mechanisms of proteolysis of SARS-CoV-2 M pro by QM/MM computational methods. *Chem. Sci.* **11**, 10626–10630 (2020).

54. S. T. Ngo, T. H. Nguyen, N. T. Tung, B. K. Mai, Insights into the binding and covalent inhibition mechanism of PF-07321332 to SARS-CoV-2 M pro. *RSC Adv.* **12**, 3729–3737 (2022).

55. K. Fan, P. Wei, Q. Feng, S. Chen, C. Huang, L. Ma, B. Lai, J. Pei, Y. Liu, J. Chen, L. Lai, Biosynthesis, purification, and substrate specificity of severe acute respiratory syndrome coronavirus 3C-like proteinase. *J. Biol. Chem.* **279**, 1637–1642 (2004).

56. W. C. Hsu, H. C. Chang, C. Y. Chou, P. J. Tsai, P. I. Lin, G. G. Chang, Critical assessment of important regions in the subunit association and catalytic action of the severe acute respiratory syndrome coronavirus main protease. *J. Biol. Chem.* **280**, 22741–22748 (2005).

57. S. Chen, J. Zhang, T. Hu, K. Chen, H. Jiang, X. Shen, Residues on the dimer interface of SARS coronavirus 3C-like protease: Dimer stability characterization and enzyme catalytic activity analysis. *J. Biochem.* **143**, 525–536 (2008).

58. N. Díaz, D. Suárez, Influence of charge configuration on substrate binding to SARS-CoV-2 main protease. *Chem. Commun.* **57**, 5314–5317 (2021).
